# Brief Outpatient Rehabilitation Program for Post–COVID-19 Condition: A Randomized Clinical Trial

**DOI:** 10.1001/jamanetworkopen.2024.50744

**Published:** 2024-12-19

**Authors:** Tom Farmen Nerli, Joel Selvakumar, Erin Cvejic, Ingar Heier, Maria Pedersen, Tone Langjordet Johnsen, Vegard Bruun Bratholm Wyller

**Affiliations:** 1 Department of Pediatric and Adolescent Medicine, Akershus University Hospital, Lørenskog, Norway; 2 Division of Physical Medicine and Rehabilitation, Vestfold Hospital Trust, Tønsberg, Norway; 3 Institute of Clinical Medicine, University of Oslo, Oslo, Norway; 4 Sydney Health Literacy Lab, Sydney School of Public Health, Faculty of Medicine and Health, University of Sydney, New South Wales, Australia; 5 Department of Pediatric and Adolescent Medicine, Oslo University Hospital, Rikshospitalet, Norway; 6 Department of Health, Social and Welfare Studies, University of South-Eastern Norway, Horten, Norway

## Abstract

**Importance:**

Post–COVID-19 condition (PCC) is emerging as a common and debilitating condition with few treatment options.

**Objective:**

To assess the effectiveness of a brief outpatient rehabilitation program based on a cognitive and behavioral approach for patients with PCC.

**Design, Setting, and Participants:**

Patients with mild to moderate PCC were randomized 1:1 to an established transdiagnostic rehabilitation program or care as usual at a single referral center recruiting from the region of the South-Eastern Norway Regional Health Authority. Participants were followed up after treatment completion and 12 months after enrollment using participant-reported outcome measures. Data were collected from February 22, 2022, until April 15, 2024. Intention-to-treat analysis was performed.

**Intervention:**

The program consisted of 2 to 8 outpatient encounters with approximately 2 to 6 weeks between each encounter. The intervention was theoretically grounded in the cognitive activation theory of stress, and physicians and physiotherapists were trained in cognitive and behavioral approaches with targeted negative stimuli and response outcome expectancies being particularly important.

**Main Outcomes and Measures:**

Participant-reported physical function assessed by the Short-Form Health Survey 36 Physical Function Subscale (SF-36-PFS) served as the primary outcome. Secondary outcome measures were the remaining subscales of the SF-36, return to work self-efficacy and symptom scores on fatigue, postexertional malaise, breathlessness, cognitive difficulties, sleep problems, anxiety and depression symptoms, and smell and taste abnormalities. Safety measures included primary health care contacts; hospital admissions; initiation of pharmacologic and/or nonpharmacologic therapy; occurrence of novel disease, illness, or other health problems; worsening of selected key symptoms; working abilities; and thoughts of suicide.

**Results:**

A total of 473 patients with mild to moderate PCC were assessed for eligibility (n = 364 physician referred; n = 109 self-referred); 314 were included (225 females [72%]; mean [SD] age, 43 [12] years) and 231 completed the primary end point evaluation. The SF-36-PFS scores improved statistically and clinically significantly in the intervention group (score difference between groups, 9.2; 95% CI, 4.3-14.2; *P* < .001; Cohen *d* = 0.43; intention-to-treat analysis). The effect was sustained over time. Most secondary and safety measures favored the intervention.

**Conclusions and Relevance:**

In this randomized clinical trial, a brief outpatient rehabilitation program with a cognitive and behavioral approach in patients with PCC was effective and safe. This trial adds to the evidence supporting such interventions in routine clinical care. Future research should investigate which elements of this approach are the most effective and identify subgroups for which the current treatment is most relevant.

**Trial Registration:**

ClinicalTrials.gov Identifier: NCT05196451

## Key Points

**Question:**

Is brief outpatient rehabilitation based on a cognitive and behavioral approach more effective than care as usual for improving physical function in individuals with post–COVID-19 condition (PCC)?

**Findings:**

In this randomized clinical trial of 314 patients with PCC, self-reported physical function improved statistically and clinically significantly in the intervention group after 2 to 8 outpatient encounters. The effect was sustained over time and adverse effects were negligible.

**Meaning:**

The findings of this trial suggest that brief outpatient rehabilitation based on a cognitive and behavioral approach is effective and safe for patients with PCC.

## Teaser

This randomized clinical trial examines the effectiveness of a 2- to 6-week outpatient cognitive and behavioral rehabilitation program for individuals with post–COVID-19 condition.

## Introduction

Post–COVID-19 condition (PCC) is defined by the persistence of symptoms such as fatigue, dyspnea, and cognitive dysfunction for 3 months or longer following an infection with SARS-CoV-2 combined with major functional impairments and no other condition explaining the symptoms.^1^ Evidence suggests that PCC is a variant of postinfective fatigue syndrome (PIFS), which is a common sequela across several infectious diseases.^2,3,4^ Post–COVID-19 condition is a substantial burden for patients, their caretakers, the health care system, and society in general.^5,6^ Prevalence estimates vary; controlled studies report 6.6% to 45%.^7,8^

Treatment options for PCC are scarce due to limited knowledge of its pathophysiologic characteristics. Several potential mechanisms have been put forward,^9,10^such as endothelial dysfunction,^11^ autoimmune reactions, persistent viral reservoirs or inflammation,^12,13^ and direct tissue damage.^14^ While most studies have focused on biological mechanisms, there is also evidence that psychosocial factors play a role. Factors such as loneliness,^15^ neuroticism, worrying tendencies, depression, anxiety, and psychological distress^2,16,17,18,19,20,21,22^ have been associated with persistent post–COVID-19 symptoms and disability. In PIFS, subconscious expectancies, worrying tendencies, and associative learning processes have been proposed as perpetuating factors.^23,24,25^ It is therefore conceivable that cognitive and/or behavioral interventions targeting perpetuating factors described in the cognitive activation theory of stress (CATS) may have a beneficial effect. Previous research has suggested that cognitive behavioral therapy (CBT) designed for PIFS and PCC^4,26,27^ as well as other behavioral interventions^28,29^ can lead to improvement of fatigue and functional capacity. Potential risk of becoming worse and potential unsuitability for individuals with severe symptoms have also been reported.^30^ Still, well-designed clinical trials are few, and evidence for therapeutic interventions that can inform clinical guidelines worldwide is lacking.^31,32^

We performed a pragmatic randomized clinical trial with 12 months’ follow-up to investigate the effectiveness of a brief outpatient rehabilitation program using a cognitive and behavioral approach based on the CATS for patients with PCC. The pragmatic component refers to an approach in which the intervention was fully embedded in the daily work and routines of an outpatient clinic. We hypothesized that the program would have a long-lasting beneficial effect on functional capacity and symptoms compared with care as usual (CAU), with no adverse effects.

## Methods

### Study Design

This was a 2-arm, pragmatic randomized clinical trial embedded in routine clinical care at 1 center. Assessments were performed at enrollment (T0, before randomization), immediately after completion of the intervention (T1), and 12 months after enrollment (T2). The primary outcome measure was participant-reported physical function at T1. The trial was approved by the Regional Committee for Ethics in Medical Research and followed the Consolidated Standards of Reporting Trials (CONSORT) reporting guideline. All patients gave written informed consent; financial compensation was not provided. The protocol is available is Supplement 1 and the statistical analysis plan is available in Supplement 2.

Participants were randomized in a 1:1 ratio to either the intervention or CAU group. Randomization was performed with REDCap software (Research Electronic Data CaptureA), using block size that varied randomly between 4 and 6. Allocation concealment was ensured using the sequentially numbered, opaque, sealed envelope technique. Due to the nature of the intervention, participants, therapists, and study assistants were not blinded to randomization outcome. However, blinding was ensured during analysis of the effectiveness variables.

### Participants

Patients with PCC were recruited from February 22, 2022, to March 31, 2023, via self-referral or physician referral. Inclusion criteria were (1) age 16 years or older, (2) confirmed acute COVID-19 by positive polymerase chain reaction or rapid antigen test, (3) persistent symptoms for at least 3 months following the acute infection without a symptom-free interval, and (4) functional disability to an extent that interrupts all or most normal activities. Exclusion criteria were (1) other chronic illness or demanding life situations that might explain persistent symptoms and disability; (2) sustained organ damage, such as heart and lung damage, post–intensive care syndrome, critical illness, and other severe neurologic disorders, excluding anosmia and ageusia, following acute, serious COVID-19; (3) being bedridden; and (4) insufficient command of the Norwegian language. Eligibility screening was performed by telephone contact with a research assistant. The final decision on enrollment was made by a physician (T.F.N.) based on a brief clinical interview and examination.

### Intervention

The intervention consisted of 2 to 8 outpatient encounters with approximately 2 to 6 weeks between the encounters. The intervention was identical to the study site’s routine clinical approach toward patients with persistent physical symptoms, and no extra resources were provided.

The intervention was theoretically grounded in the CATS.^25^ In brief, CATS states that any stressful event (psychologically as well as biologically) necessitates an adaptive response, which may imply bodily symptoms.^25^ Normally, the adaptive response is brief and self-limited. However, a sustained response may have disadvantageous effects and result in a wide variety of bodily symptoms. Cognitive factors, such as subconscious expectancies, are key determinants of the degree and duration of the adaptive response and are themselves shaped by individual learning history. Of particular importance are the stimuli expectancies influenced by classical conditioning and response outcome expectancies that occur with operant conditioning.^33^ Altering these expectancies is thus the purpose of the intervention.

The rehabilitation program was structured as a 2-stage process with the overall aim of restoring physical function. At the first stage, a physician conducted a clinical examination and addressed symptoms, functional impairments, and previously performed examinations intending to (1) rule out differential diagnoses, (2) validate the symptoms as real experiences, and (3) provide a psychoeducative explanation within the CATS theoretical framework. The latter always included an explanation of normal responses to stressful situations, emphasizing that moderate stress may promote thriving, and how certain infections (eg, COVID-19) could trigger maladaptive responses and diverse, unpredictable, and bothersome symptoms (eg, fatigue, dyspnea, and brain fog). Hence, the first stage provided cognitive reassurance^34^ that bodily symptoms do not necessarily indicate a disease but rather a disorder that is temporary and amendable.

At the second stage, a predefined list of 19 topics related to stimuli and response outcome expectancies functioned as a framework for in-session structure and content (eTable 1 in Supplement 3). Cognitive behavioral therapy–trained physiotherapists supervised the patients by using nondirective communication,^35^ socratic dialogue, and guided discovery,^36^ prompting patients to infer that recovery would require an active pursuit of physical and mental tasks, thereby fostering positive stimuli expectancies. The therapists also questioned patients’ perceived benefit of symptom surveillance and explained why conscious awareness of the relationship between activities and symptoms may perpetuate the latter. Patients were encouraged to explore new activities between therapy sessions, with an understanding that this was safe and necessary for improvement.^37^ It was anticipated that favorable experiences would induce coping, ie, promote positive response outcome expectancies.

### Effectiveness Outcome Measures

At baseline (T0), participants completed a composite questionnaire consisting of the following validated inventories: the Short-Form Health Survey 36 (SF-36),^38^ the Chalder Fatigue Questionnaire,^39^ the Hospital Anxiety and Depression Scale,^40^ the DePaul Symptom Questionnaire-2,^41^ the Karolinska Sleep Questionnaire,^42^ the Medical Research Council Dyspnoea Scale,^43^ and the Return-to-Work Self-Efficacy Questionnaire.^44^ The questionnaire also included 4 items addressing cognitive difficulties and 2 items addressing smell and taste abnormalities that have been used in a previous COVID-19 study,^2^ as well as questions on demographic characteristics.

All items concerning intervention effectiveness were repeated at T1 and T2. In the CAU group, T1 was matched by inclusion time point. Questionnaires were web-based and answered online. The SF-36 Physical Function Subscale (SF-36-PFS) score at T1 served as the primary outcome measure. It consists of 10 items scored on Likert scales and was recoded to achieve a 100-point scale, where higher scores reflect better physical functioning.^38^ Secondary outcome measures were the remaining subscales of the SF-36 (role limitations due to physical problems, bodily pain, general health, vitality, social functioning, role limitations due to emotional problems, and mental health), the Return-to-Work Self-Efficacy Questionnaire total score, the Chalder Fatigue Questionnaire total score, the postexertional malaise (PEM) score from DePaul Symptom Questionnaire-2, the Medical Research Council Dyspnoea Scale total score, the Karolinska Sleep Questionnaire total score, the Hospital Anxiety and Depression Scale depression subscore, the Hospital Anxiety and Depression Scale anxiety subscore, the mean score of cognitive difficulties, and the mean score of smell and taste abnormalities, as well as the SF-36-PFS score at T2. Recovery of physical function was defined as a SF-36-PFS score at the population norm^45^ (approximately 85) or higher. Therapists completed a composite questionnaire after each patient encounter recording fidelity according to a list of predefined topics on Likert scales ranging from 0 to 6, with higher scores indicating better fidelity.

### Safety Outcome Measures and Adverse Events

Safety outcome measures were defined as primary health care contacts; hospital admissions; initiation of pharmacologic and/or nonpharmacologic therapy; occurrence of novel disease, illness, or other health problems; worsening of selected key symptoms (fatigue, concentration problems, pain, and sadness); working abilities; and thoughts of suicide. These outcomes were charted by a customized web-based questionnaire using dichotomous (yes or no) or 5-point Likert scales for each item as well as optional free-text comments. The questionnaire was completed online at T1 and T2.

Questionnaire results and spontaneous patient reports were used to monitor adverse events (AEs) and serious AEs (SAEs). Adverse event was defined as any medical occurrence in the follow-up period, whether or not attributed to or considered to be causally related to the intervention. Serious AE was defined as an AE meeting any of the following criteria: deadly, life-threatening, requiring hospitalization, or resulting in a major disability. Three physicians (T.F.N., J.S., and V.B.B.W.) rated independently whether an AE or SAE could potentially have been caused by the intervention and discussed discrepancies until consensus was reached.

### Subgroup Classification

Adherence to the modified Fukuda definition of PIFS caseness,^46^ which is stricter than the current PCC definition, was operationalized as previously described (eFigure 1 in Supplement 3).^2^ Classification of participants as having or not having PIFS was performed independently by 2 physicians (T.F.N. and J.S.), and discrepancies were discussed until consensus was reached.

### Consumer Contribution

Three individuals diagnosed with PCC and receiving the routine outpatient program before the present study provided input on design in the planning stage of the project. Representatives from the patient organization Recovery Norway participated in discussions concerning the intervention, and the consumer group from the International Collaborative on Fatigue Following Infection advised on analysis and dissemination strategies.

### Statistical Analysis

A score difference of 10 points on the SF-36-PFS was considered clinically significant.^47^ Assuming an SD of 25 in the population under study^45^ and a dropout rate of 20%, a target inclusion of 310 participants would yield a power of 90% (α = .05) to detect a small to medium effect size (Cohen *d* = 0.2-0.5).

The primary analysis was an intention-to-treat analysis of all randomized participants featuring multiple imputation of missing values. Per-protocol analyses were conducted as sensitivity analyses. Usually, generalized linear models (analysis of covariance) were applied for analyses of treatment effects, with the baseline values of each effectiveness end point included as covariates; model fit was assessed by inspection of residual plots. Separate tests were conducted for all outcome variables at T1 and T2. For each statistical analysis, the net intervention effect (the mean change in the intervention group minus the mean change in the CAU group) was calculated from the parameters of the fitted analysis of covariance model and reported with 95% CI. Differential effects in subgroups were explored by including relevant interaction terms. For ordinal variables, sensitivity analyses using ordinal logistic regression were carried out. Safety data were summarized through data tabulations and descriptive statistics. Associations between prevalent symptoms were explored using Spearman rank correlation.

SPSS, version 29.0 (SPSS Inc) and R, version 4.2.3 (R Foundation for Statistical Computing) were used for all statistical analyses. All tests were 2-sided, and *P* ≤ .05 was considered statistically significant throughout. No interim analysis was carried out and no correction for multiple comparisons was applied.

## Results

### Participants and Study Validity

**Figure 1.**
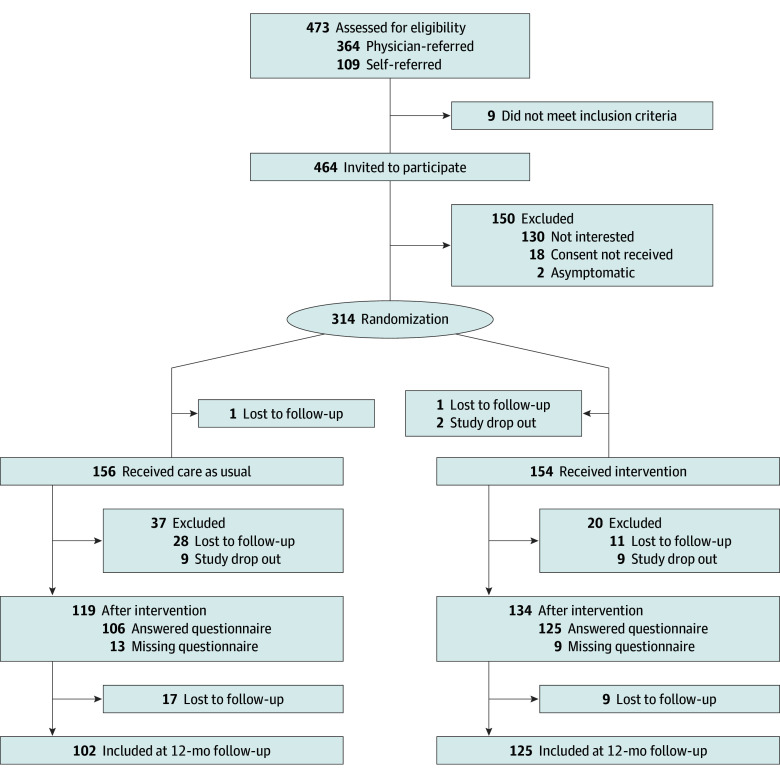
Study Design and Overview Loss to follow-up includes primary end point missing; participants with no response after baseline assessment, first follow-up after completion of the intervention (approximately 6 months after inclusion), and second follow-up approximately 6 months after completion of the intervention (approximately 12 months after inclusion); and protocol deviations. Study drop out includes participants with active withdrawal from the study.

A total of 473 patients with mild to moderate PCC were assessed for eligibility (364 physician referred, 109 self-referred), of whom 314 were included (225 females [72%]; 89 males [28%]; mean [SD] age, 43 [12] years) (Figure 1). Fatigue, cognitive difficulties, and PEM were the most prevalent symptoms (eTable 2 in Supplement 3) and correlated with each other and most other symptoms (eFigure 2 in Supplement 3). Self-referral was associated with accepting the invitation to join the study, whereas sex and age had no association with enrollment (eTable 3 in Supplement 3). A total of 310 patients started treatment (156 in the CAU group, 154 in the intervention group), 253 patients completed treatment (231 with data on the primary outcome measure), and 227 patients completed the entire follow-up period, resulting in a total of 35% of individuals having incomplete data (Figure 1). Active treatment withdrawal in the intervention group was 6%, and 2 of the 9 participants who withdrew actively reported the treatment as the reason for dropping out. Analysis of missing data points revealed a missing completely at random pattern (*P* = .40; Little Missing Completely at Random test). Allocation to CAU and lower age were associated with loss to follow-up (eTable 4 in Supplement 3). At inclusion, a total of 107 participants (35%) adhered to the modified Fukuda definition of PIFS.^46^ All variables were almost equally distributed across the 2 allocation groups except sex, with the proportion of females being higher in the intervention group than in the CAU group (Table 1). Fidelity scores assessed during the intervention period were generally high among the involved therapists (eTable 1 in Supplement 3). A total of 158 protocol deviations were noted, of which the most common were loss to follow-up and primary end point missing (eTable 5 in Supplement 3).

**Table 1.** Study Population Characteristics

Characteristic	Population, No. (%)	
	Care as usual	Intervention
Allocation	157 (50)	157 (50)
Demographic		
Sex		
Female	103 (66)	122 (78)
Male	54 (34)	35 (22)
Age, mean (SD), y	42 (12)	43 (12)
BMI, mean (SD)	26 (4.8)	26 (4.4)
Norwegian ethnicity	147 (94)	143 (93)
Highest level of education		
Secondary school	7 (4.5)	3 (1.9)
High school	21 (14)	24 (16)
Apprenticeship	32 (21)	26 (17)
University, lower degree	56 (36)	65 (42)
University, higher degree	29 (25)	33 (21)
Other, NA	1 (0.6)	3 (1.9)
Comorbidities^a^		
None	93 (60)	94 (61)
Asthma, allergy, and atopy	27 (17)	27 (17)
Psychological	5 (3.2)	6 (3.8)
Migraine and headache syndromes	7 (4.5)	7 (4.5)
Cardiovascular	8 (5.1)	8 (5.1)
Endocrinologic	7 (4.5)	9 (5.7)
Gynecologic	5 (3.2)	2 (1.3)
Gastroenterologic	12 (7.6)	5 (3.2)
Pain syndromes	4 (2.5)	6 (3.8)
Other	12 (7.6)	13 (8.3)
Regular use of tobacco products	32 (21)	35 (23)
COVID-19 and vaccination		
Diagnosis of initial acute COVID-19		
PCR, medical laboratory	86 (55)	96 (61)
Rapid antigen test	69 (44)	60 (38)
Symptomatic close contact	2 (1.3)	1 (0.6)
Hospitalized during acute COVID-19	5 (3.2)	4 (2.5)
Time since initial acute COVID-19, mean (SD), d	266 (185)	244 (154)
Fulfilling case definition of PIFS	53 (34)	54 (35)
Vaccine against SARS-CoV-2		
None	9 (5.8)	6 (3.9)
1 Dose	9 (5.8)	11 (7.1)
2 Doses	62 (40)	54 (35)
3 Doses	76 (49)	83 (54)
Outcome variables, SF-36 subscores, mean (SD)^b^		
Physical function	63 (20)	63 (23)
Role limitations due to physical problems	7.1 (20)	8.1 (21)
Bodily pain	49 (10)	49 (10)
General health	44 (22)	45 (22)
Vitality	22 (15)	22 (15)
Social functioning	40 (25)	41 (26)
Role limitation due to emotional problems	58 (44)	53 (45)
Mental health	63 (16)	64 (19)
Outcome variables, working abilities, mean (SD)		
Return to work self-efficacy^c^	2.8 (1.0)	2.9 (0.9)
Outcome variables, symptoms, mean (SD)^b^		
Fatigue	25 (5.2)	25 (4.5)
Postexertional malaise	65 (24)	66 (24)
Breathlessness	1.1 (0.9)	1.1 (0.9)
Cognitive difficulties	3.4 (1.0)	3.2 (1.0)
Sleep problems	36 (9.4)	36 (8.7)
Anxiety symptoms	8.0 (4.3)	7.3 (4.5)
Depressive symptoms	7.2 (4.0)	6.7 (4.1)
Smell and/or taste abnormalities	2.2 (1.5)	1.9 (1.4)
Study design characteristics, mean (SD)		
Time from T0 to T1, d	200 (75)	202 (83)
Time from T0 to T2, d	396 (37)	392 (38)
No. of outpatient appointments, intervention group, median (range)	NA	4 (0-8)
Follow-up for PCC between T0 and first T1^d^		
None	14 (13)	35 (28)
General practitioner	92 (87)	90 (72)
Specialist physician (outpatient)	29 (27)	20 (16)
Physical therapy	32 (30)	25 (20)
Psychotherapy/counseling	17 (16)	7 (6)
Inpatient rehabilitation	18 (17)	0

Abbreviations: BMI, body mass index (calculated as weight in kilograms divided by height in meters squared); NA, not applicable; PCC, post–COVID-19 condition; PCR, polymerase chain reaction; PIFS, postinfective fatigue syndrome; SF-36, Short-Form Health Survey 36; T0, baseline; T1, first follow-up; T2, second follow-up.

^a^ The comorbidities were not sufficient to exclude participants from the trial.

^b^ All survey, scale, and questionnaire information (item count, score range, and meaning of higher scores) appears in the footnotes to Table 2.

^c^ Based on 11 single items from the Return-to-Work Self-Efficacy scale^44^; total range, 1 to 6, where higher scores indicate high work-related self-efficacy.

^d^ Data available for 106 participants in the care-as-usual group for this variable. One individual may have received follow-up from more than 1 of the alternatives.

### Effectiveness Outcomes

**Figure 2.**
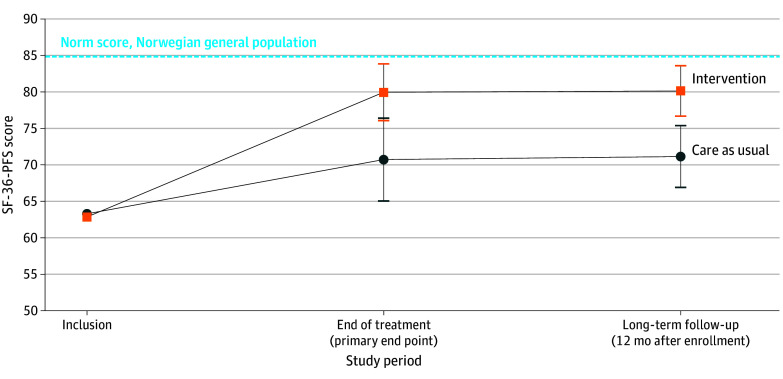
Intervention Effect on the Primary Outcome, Intention-to-Treat Analysis Featuring Multiple Imputation End of treatment was the primary end point. Dots and squares represent estimated marginal means; error bars indicate 95% CIs, using analysis of covariance modeling. SF-36-PFS indicates Health Survey 36 Physical Function Subscale.

Data were collected from February 22, 2022, until April 15, 2024. Self-reported physical function on the SF-36-PFS at T1 (primary outcome) improved significantly more in the intervention group than in the CAU group (score difference, 9.2; 95% CI, 4.3-14.2; *P* < .001; Cohen *d* = 0.43; intention-to-treat analysis) (Figure 2; eFigure 3 in Supplement 3; Table 2). The difference between the 2 groups was almost identical at long-term (T2) analysis, indicating a sustained effect. The results pertaining to most secondary outcome measures were in favor of the intervention group. Smell and/or taste difficulties and bodily pain were the only outcome measures with no statistically significant effect at any time point. Role limitations due to physical problems, breathlessness, and sleep problems did not show significant effect at T1 but did at T2. The Cohen *d* values varied from 0.26 to 0.51 among the statistically significant differences, corresponding to small to moderate effect sizes. Results of the per-protocol analyses strongly resembled those of the intention-to-treat analyses (eTable 6 and eTable 7 in Supplement 3), but the intervention effects tended to be stronger, with Cohen *d* values ranging from 0.33 to 0.71. A total of 17% of the individuals in the intervention group and 20% in the CAU group met the recovery threshold, defined as SF-36-PFS score at the population norm or higher at baseline, while 50% of individuals in the intervention group and 32% of those in the CAU group met the criteria at T1 (eFigure 3, eTable 8 in Supplement 3). Explorative subgroup analyses revealed few differential outcomes related to PIFS diagnosis (eTable 9 in Supplement 3); however, for social functioning, the beneficial intervention effect was somewhat stronger in the PIFS subgroup at T2 (interaction *P* = .02). Differential outcomes related to PEM scores at baseline showed significantly stronger beneficial intervention effect in the high PEM group (defined as upper quartile) for cognitive difficulties and anxiety (T1 and T2) as well as bodily pain and fatigue (T2) (eTable 10 in Supplement 3).

**Table 2.** Outcome of the Intervention; Intention-to-Treat Analyses^a^

Outcome	Measure				
	Care as usual, mean (SEM)	Intervention, mean (SEM)	Difference (95% CI)	*P* value	Cohen *d*
**Primary end point, SF-36 subscore**					
Physical function^b^					
T1	70.7 (2.8)	80.0 (2.0)	9.2 (4.3 to 14.2)	<.001	0.43
T2	71.1 (2.1)	80.1 (1.8)	9.0 (4.0 to 13.9)	<.001	0.42
**Secondary end point, SF-36 subscores**					
Role limitations due to physical problems^c^					
T1	21.9 (6.0)	31.7 (4.8)	9.8 (−0.5 to 20.1)	.06	0.25
T2	24.5 (5.3)	39.4 (3.8)	14.9 (3.6 to 26.2)	.01	0.36
Bodily pain^d^					
T1	51.4 (1.7)	51.5 (1.3)	0.08 (−3.0 to 3.2)	.96	0.01
T2	51.6 (1.4)	53.9 (1.2)	2.4 (−1.0 to 5.8)	.17	0.22
General health^e^					
T1	45.0 (4.2)	54.3 (2.9)	9.3 (3.3 to 15.3)	.003	0.40
T2	44.6 (2.6)	52.2 (2.1)	7.6 (1.2 to 13.9)	.02	0.30
Vitality^f^					
T1	33.7 (3.1)	40.5 (2.2)	6.7 (1.3 to 12.2)	.02	0.31
T2	29.9 (2.5)	37.6 (1.8)	7.6 (2.3 to 13.0)	.005	0.37
Social functioning^g^					
T1	46.0 (4.2)	60.4 (3.2)	14.4 (7.4 to 21.5)	<.001	0.52
T2	52.8 (2.7)	66.8 (2.3)	14.0 (7.2 to 20.8)	<.001	0.51
Role limitations due to emotional problems^h^					
T1	61.9 (7.3)	62.8 (5.0)	0.9 (−12.6 to 14.4)	.90	0.02
T2	53.0 (5.3)	70.4 (4.3)	17.4 (4.4 to 30.4)	.009	0.36
Mental health^i^					
T1	66.7 (2.3)	71.6 (1.5)	4.9 (0.9 to 8.9)	.02	0.31
T2	66.3 (1.3)	72.9 (1.1)	6.6 (3.3 to 9.9)	<.001	0.43
**Secondary end point, working abilities**					
Return to work self-efficacy^j^					
T1	3.2 (0.15)	3.7 (0.12)	0.5 (0.2 to 0.8)	.001	0.37
T2	3.5 (0.13)	3.9 (0.12)	0.4 (0.1 to 0.7)	.005	0.33
**Secondary end point, symptoms**					
Fatigue^k^					
T1	21.2 (1.2)	18.8 (0.9)	−2.4 (−4.3 to −0.5)	.01	0.33
T2	20.0 (0.8)	17.6 (0.6)	−2.4 (−4.2 to −0.7)	.008	0.35
Postexertional malaise^l^					
T1	50.4 (4.3)	37.9 (3.2)	−12.5 (−19.6 to −5.4)	.001	0.42
T2	51.7 (3.0)	39.2 (2.6)	−12.4 (−19.8 to −5.1)	.001	0.41
Breathlessness^m^					
T1	1.0 (0.15)	0.7 (0.10)	−0.2 (−0.5 to −0.04)	.10	0.22
T2	1.0 (0.10)	0.7 (0.08)	−0.4 (−0.6 to −0.2)	.001	0.42
Cognitive difficulties^n^					
T1	3.3 (0.12)	2.9 (0.11)	−0.4 (−0.7 to −0.2)	.001	0.40
T2	3.1 (0.11)	2.8 (0.08)	−0.3 (−0.5 to −0.1)	.02	0.28
Sleep problems^o^					
T1	39.6 (1.3)	41.6 (1.0)	1.9 (−0.5 to 4.3)	.12	0.19
T2	38.0 (1.2)	42.8 (0.8)	4.8 (2.3 to 7.4)	<.001	0.45
Anxiety symptoms^p^					
T1	6.7 (0.27)	5.9 (0.26)	−0.8 (−1.5 to −0.01)	.05	0.20
T2	7.2 (0.26)	6.2 (0.26)	−0.9 (−1.6 to −0.2)	.01	0.26
Depressive symptoms^q^					
T1	6.1 (0.26)	4.9 (0.25)	−1.2 (−1.9 to −0.5)	.001	0.33
T2	6.1 (0.27)	4.9 (0.26)	−1.2 (−1.9 to −0.5)	.001	0.34
Smell and/or taste abnormalities^r^					
T1	1.9 (0.17)	1.8 (0.13)	−0.1 (−0.4 to 0.2)	.49	0.08
T2	2.0 (0.13)	1.9 (0.11)	−0.1 (−0.4 to 0.2)	.43	0.09

Abbreviations: SF-36, Short Form Health Survey 36; T1, first follow-up, after completion of the intervention (approximately 6 months after inclusion); T2, second follow-up, approximately 6 months after completion of the intervention (approximately 12 months after inclusion).

^a^ The primary analysis was an intention to-treat analysis of all randomized participants featuring multiple imputation of missing values. For each statistical analysis, the net intervention effect (the mean change in the intervention group minus the mean change in the usual care group) was calculated from the parameters of the fitted analysis of covariance model and reported with 95% CIs.

^b^ Based on 10 single items from the SF-36^38^; total range, 0 to 100 where higher scores indicate better physical function.

^c^ Based on 4 single items from the SF-36^38^; total range, 0 to 100, where higher scores indicate fewer limitations due to physical problems.

^d^ Based upon 2 single items from the SF-36^38^; total range, 0 to 100, where higher scores indicate less pain.

^e^ Based on 5 single items from the SF-36^38^; total range, 0 to 100, where higher scores indicate better general health.

^f^ Based on 4 single items from the SF-36^38^; total range, 0 to 100, where higher scores indicate better vitality.

^g^ Based on 2 single items from the SF-36^38^; total range, 0 to 100, where higher scores indicate better social functioning.

^h^ Based on 3 single items from the SF-36^38^; total range, 0 to 100, where higher scores indicate fewer limitations due to emotional problems.

^i^ Based on 5 single items from the SF-36^38^; total range, 0 to 100, where higher scores indicate better mental health.

^j^ Based on 11 single items from the Return-to-Work Self-Efficacy scale^44^; total range, 1 to 6, where higher scores indicate high work-related self-efficacy.

^k^ Based on 11 single items from the Chalder Fatigue Scale^39^; total range, 0 to 33, where higher scores indicate more fatigue.

^l^ Based on 5 single items from the DePaul Symptom Questionnaire-2^41^ addressing the frequency of symptoms; total range, 0 to 100, where higher scores indicate more postexertional malaise.

^m^ Based on 1 item from the Medical Research Council Dyspnoea Scale^43^; total range, 0 to 4, where higher scores indicate more dyspnea.

^n^ Based on the average of 4 single items addressing memory, concentration, confusion, and the ability to take decisions used in a previous COVID-19 cohort study^2^; total range, 1 to 5, where higher scores imply more cognitive difficulties.

^o^ Based on 12 single items from the Karolinska Sleep Questionnaire^42^ addressing the frequency of sleep-related problems; total range, 12 to 72, where higher scores indicate better sleep.

^p^ Based on 7 single items from the Hospital Anxiety and Depression Scale anxiety subscale^40^; total range, 0 to 21, where higher scores indicate more symptoms related to anxiety.

^q^ Based on 7 single items from the Hospital Anxiety and Depression Scale depression subscale^40^; total range, 0 to 21, where higher scores indicate more symptoms related to depression.

^r^ Based on the average of 2 single items addressing smell and taste abnormalities used in a previous COVID-19 cohort study^2^; total range, 1 to 5, where higher scores imply more smell and taste abnormalities.

### Safety Outcomes

Self-reported health status was better in the intervention group than in the CAU group for most items at both time points, except for hospital admissions and occurrence of novel disease at T2 (Table 3). Also, the number of AEs and SAEs was lower in the intervention group (eTable 11 in Supplement 3). A total of 4 SAEs in the intervention group were recorded; 1 might have been due to diagnostic delay, but none of the others were considered to be causally related to the intervention. A total of 9 individuals in the intervention group experienced a decrease in self-reported physical function from T0 to T1 compared with 25 in the CAU group (eFigure 3, eTable 12 in Supplement 3), and a total of 14 individuals in the intervention group experienced an increase in PEM from T0 to T1 compared with 31 in the CAU group (eTable 13 in Supplement 3).

**Table 3.** Safety Variables, Alteration of Health Status Since Previous Study Visit^a^

Variable	No. (%)			
	T1		T2	
	Care as usual (n = 106)	Intervention (n = 125)	Care as usual (n = 102)	Intervention (n = 125)
Appointment with general practitioner/other primary care service	92 (87)	90 (72)	78 (76)	81 (65)
Hospital admission^b^	6 (6)	4 (3)	2 (2)	7 (6)
Other health care contacts^c^	96 (90)	52 (42)	82 (80)	62 (50)
Specialist physician (outpatient)	29 (27)	20 (16)	18 (18)	22 (18)
Physical therapy, etc	32 (30)	25 (20)	31 (30)	25 (20)
Psychotherapy/counseling, etc	17 (16)	7 (6)	13 (13)	11 (9)
Inpatient rehabilitation	18 (17)	NA	20 (20)	4 (3)
Initiation of pharmacologic therapy	35 (33)	24 (19)	31 (30)	27 (22)
Initiation of nonpharmacologic therapy	25 (24)	13 (10)	15 (15)	11 (9)
Occurrence of novel diseases, illnesses, other health problems^d^	36 (34)	40 (32)	39 (38)	50 (40)
Increased experience of fatigue	19 (18)	13 (10)	23 (23)	16 (13)
Increased experience of concentration problems	18 (17)	18 (14)	18 (18)	15 (12)
Increased experience of pain	15 (14)	13 (10)	13 (13)	11 (9)
Increased experience of sadness	21 (20)	14 (11)	24 (24)	17 (14)
Increased ability to work	41 (39)	73 (58)	29 (28)	58 (46)
Any thoughts of committing suicide	11 (10)	11 (9)	12 (12)	7 (6)

Abbreviations: T1, first follow-up, after completion of the intervention (approximately 6 months after inclusion); T2, second follow-up, approximately 6 months after completion of the intervention (approximately 12 months after inclusion).

^a^ All safety variables are based on a separate, self-composed questionnaire featuring partly dichotomous response scales (yes/no) and partly 1 to 5 Likert response scales with optional free-text comments, where scores 1 to 3 indicate improved or unaltered health status and scores 4 to 5 indicate poorer health status. The latter responses were dichotomized prior to tabulation. Free-text comments were used to better differ between the domains. The patients might have multiple other health care contacts and/or occurrence of novel disease.

^b^ Hospital admission reflects serious adverse events reported in eTable 11 in Supplement 3.

^c^ Categorization in subgroups based on free-text comments.

^d^ Mainly composed of viral upper respiratory tract infections.

## Discussion

This randomized clinical trial found that patients with PCC reported sustained and clinically significant better physical function following a brief outpatient rehabilitation program based on a cognitive and behavioral approach compared with CAU. Other measures of functional capability, as well as symptom scores, improved significantly more in the intervention group with small to moderate effect sizes, whereas frequencies of AEs were lower.

These findings are in line with a previous randomized clinical trial investigating CBT for PCC, which also showed improved physical and social functioning and symptom scores and had comparable effect sizes.^26^ Other behavioral interventions for PCC,^29,48^ as well as another study investigating CBT for PIFS,^4^ followed the same pattern. A novel finding of the present study is that rather few outpatient encounters (2-8 per patient) were sufficient to achieve similar effects as in studies of far more resource-demanding rehabilitation programs, and with sustained effect 6 months after intervention completion. Further studies should scrutinize mediators of treatment effect such as the different components of our rehabilitation program. The favorable outcome for the intervention group on safety measures was reassuring given the frequent criticism against CBT-based treatment approaches for PCC and related disorders.^49^

Generally, there was a tendency toward stronger beneficial intervention effects among the subgroup with the highest PEM scores at baseline and those fulfilling PIFS criteria where PEM was regarded as a hallmark of PIFS.^50^ A recent review article advised against rehabilitation based on graded exercise and CBT in patients with PCC and patients with PEM.^9^ In the present study, no direct precautions were taken in regard to PEM; patients were always advised to continue exploring activities. Type of activity was not standardized, but each participant was able to choose the activity and homework based on their values, goals, and interests, as long as the activity was suitable, feasible, and enjoyable. Graded exercise was an opportunity for those who wanted it. Based on the present results, which also showed an overall significant decrease in PEM in the intervention group, it does not seem relevant to take PIFS diagnosis or the presence of PEM into account when developing an individualized rehabilitation plan. This notion fits well with another study showing that exercise is well tolerated independent of PEM in patients with PCC.^51^

Post–COVID-19 condition and PIFS are phenotypically similar to chronic fatigue syndrome,^52^ and a recent meta-analysis reported high acceptance of CBT for patients with chronic fatigue syndrome, with low rates of noncompletion, drop out, and treatment refusal.^53^ Even though 35% of patients in this study had incomplete data, active treatment withdrawal in the intervention group was 6%, and only 2 of the 9 participants who withdrew actively reported the treatment as the reason for dropping out.

The pragmatic design of the present study, including the wide inclusion criteria, makes it relevant for general practitioners. A recent study of a very similar intervention in a primary health care context showed beneficial effects for people with persistent physical symptoms, including fatigue, pain, and brain fog.^54^

### Strengths and Limitations

Strengths of the study include the pragmatic approach where the intervention was fully embedded in the daily work and routines of an outpatient clinic. This increases external validity^55^ and makes the results applicable to a general population with PCC in need of rehabilitation. Furthermore, the sample size was larger than similar intervention studies on PCC and PIFS, and the primary outcome measure (SF-36-PFS) is generally recognized as a valid and reliable outcome measure for intervention trials in the field.^56^

Limitations include the single-center approach and the lack of blinding for group allocation due to the nature of the intervention, which implies that the placebo effect cannot be controlled for. Furthermore, patients were moderately impaired and almost exclusively nonhospitalized, and the results may thus not be generalizable to patients with more disabling sequelae following severe, acute COVID-19. Similarly, current definitions of PCC are broad and based on clinical attributes alone; future identification of subgroups might elucidate which groups the current treatment is most relevant for, either alone or with treatments targeting core pathophysiologic mechanisms. The attention given to patients in the intervention group was not matched with that of the CAU group; still, most patients in the CAU group received medical care that can affect patient-reported outcome measures,^31,57^ conceivably reducing the impact of not including a sham intervention in the control group. Although no statistical evidence indicates nonrandom missingness, the possibility cannot be ruled out, particularly given the noticeable differences in missing data proportions between the intervention and CAU groups. Objective measures of physical and social function (eg, steps per day and work attendance) were not included but could have yielded valuable information; however, as the diagnosis of PCC is based on patients’ self-report, patient-reported outcomes appear to be most relevant for evaluation of a rehabilitation program.

## Conclusions

In this randomized clinical trial of patients with PCC, a brief outpatient rehabilitation program with a cognitive and behavioral approach was effective and safe. This trial adds to the evidence supporting such interventions in routine clinical care. Future research should explore which elements of this approach are the most effective and identify subgroups for whom the current treatment is most relevant.

## Notes

Supplement 1.
Trial Protocol

Supplement 2.
Statistical Analysis Plan

Supplement 3. **eTable 1.** Content Topics and Fidelity to Content Topics in All Consultations (n=516) at Stage Two of the Rehabilitation Program **eTable 2.** Symptom Prevalence at Baseline **eTable 3.** Attritions **eTable 4.** Lost to Follow-Up **eTable 5.** Protocol Deviations **eTable 6.** Sensitivity Analysis 1 **eTable 7.** Sensitivity Analysis 2 **eTable 8.** Participants Meeting Recovery Threshold of Self-Reported Physical Function, per-Protocol Data **eTable 9.** Subgroup Analyses According to PIFS Caseness at Inclusion **eTable 10.** Subgroup Analyses According to PEM Score at Inclusion **eTable 11.** Adverse Events **eTable 12.** Decrease in SF-36 Physical Function Subscore From Baseline to T1 in the Intervention Group, Qualitative Evaluation **eTable 13.** Increase in Post Exertional Malaise With DePaul Symptom Questionnaire Addressing Frequency of Symptoms From Baseline to T1 in the Intervention Group, Qualitative Evaluation **eFigure 1.** Adherence to the Modified Fukuda-Definition of PIFS Caseness **eFigure 2.** Correlation (Spearman’s Rho) Heatmap of Symptom-End Points and Selected Symptoms **eFigure 3.** Physical Function Score (0-100) and Trajectories of Participants Who Attended Any Follow-Up Timepoint
eReferences

Supplement 4.
Data Sharing Statement
