# Supplementary material for: Brief Outpatient Rehabilitation Program for Post–COVID-19 Condition: A Randomized Clinical Trial
Source: JAMA Netw Open. 2024 Dec 19;7(12):e2450744. doi: 10.1001/jamanetworkopen.2024.50744 (PMC11659907; doi:10.1001/jamanetworkopen.2024.50744)
Supplement: Supplement 1. — Trial Protocol [file jamanetwopen-e2450744-s001.pdf]

Short-time behavioural intervention in post-Covid syndrome (SIPCOV): A pragmatic randomised controlled trial

NCT05196451

February 21., 2022

Amendment #1, Mars 18., 2022: Addition of questionnaires

1. Introduction

Persistent post-infectious symptoms (PPIS) after Covid-19, often referred to as *post-Covid syndrome*, may be considered as an epidemic in its own right

The Covid-19 pandemic is an unprecedented threat to health and welfare globally. It is increasingly clear that Covid-19 may result in disabling long-term sequelae often referred to as *post-Covid syndrome* or "*Long Covid*", being equally common among hospitalized and non-hospitalized patients.[29,30] Evidence suggests that most post-Covid syndrome cases fit within the label of *Persistent post-Infectious Symptoms (PPIS)*, encompassing chronic fatigue, pain and other symptoms, but with scarce findings on standard clinical examination.[6,24,27,31]

PPIS is a common sequel after several acute infections with a diverse array of pathogens, ranging from infectious mononucleosis (IM) caused by Epstein-Barr virus, to Q-fever caused by the bacterium *Coxiella burnetii*, and gastroenteritis caused by the parasite *Giardia lamblia*,[10,12] or as an adverse effect following vaccination.[9] PPIS is a pathogen-independent phenomenon that phenotypically appears as an abnormal perpetuation of the acute sickness response (ASR), which is a stereotyped collection of physiological, behavioral and psychological manifestations to an infectious event.[1] Across multiple prospective cohort studies, almost one half of subjects report PPIS six months after the infectious event, and 10-15 % satisfy diagnostic criteria for chronic fatigue syndrome (CFS) (often also referred to as myalgic encephalomyelitis (ME)).[5,10,12,23,28] Although late recovery may occur chronic disability is common, with strong negative impact upon employment prospects and family networks, and with high societal costs.[13] Given the total number of Covid-19 cases globally (approximately 140 million by April 2021),[37] *post-Covid syndrome may be considered as an epidemic in its own right* (Fig. 1).

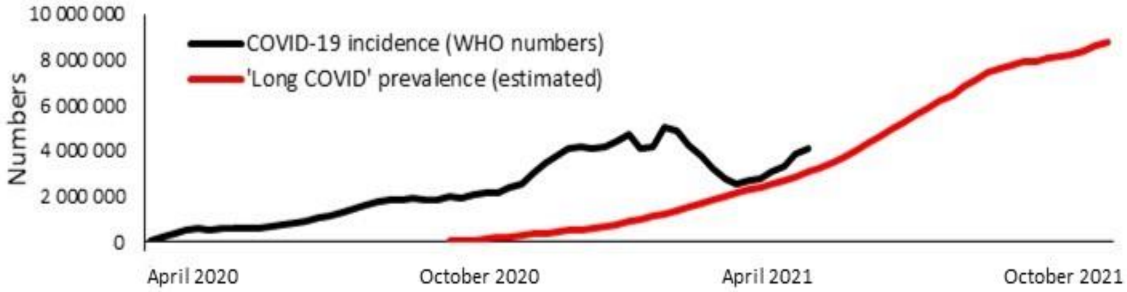

Figure 1. Covid-19 weekly incidence[37] and a conservative estimate of post-Covid syndrome prevalence (based on 10 % and 5 % case rate, respectively, 6 and 12 months after the acute infection).[12] Recent evidence suggests that the numbers may be even higher.[35]

Functional brain alterations may be a central disease mechanism of post-Covid syndrome

The underlying disease mechanisms of post-Covid syndrome remain poorly understood. However, studies of PPIS following other infections have revealed a complex blend of genetic/epigenetic[25] and psychosocial[7] vulnerabilities, in concert with autonomic,[40,41] hormonal,[22] and immunological[21] alterations.

A prominent model of PPIS consider *functional brain alterations* to be a central pathophysiological element, possibly explaining other documented aberrations.[11,26,42] This assumption is supported by functional brain imaging studies,[38,39] clinical trial outcomes,[17,32] and fundamental neurobiological concepts,[3,18] and is also intimately related to more comprehensive theories on sustained activation of biological stress responses.[33,42]

A rehabilitation program aiming to counteract functional brain alterations in post-Covid syndrome may be effective, but has not been systematically studied

If functional brain alteration is important in the pathogenesis of PPIS (including post-Covid syndrome), it is conceivable that mental and behavioural interventions may have a beneficial effect. Indeed, cognitive behavioral therapy (CBT) is of documented value in PPIS following other infections.[15,17] As for more extensive and multidisciplinary rehabilitation, little is known regarding

efficacy. To the best of our knowledge, no specific rehabilitation program has been systematically studied among post-Covid syndrome sufferers.

1.1 Needs description

The present study addresses urgent needs for the health care services as well as the scientific community

- *Patient treatment*. This project directly addresses a strong need to develop effective treatment strategies for a large patient group suffering from post-Covid syndrome disability. Thus, it significantly improve health care services. Of note, the pragmatic approach ensures strong external validity and generalizability of the results.
- *Relevance and benefit to society*. This project addresses a highly challenging health problem nationally and internationally, which may have substantial negative impact upon employment issues, social security systems, family networks etc.
- *Scientific relevance*. The project addresses a prominent knowledge gap. Also, as the intervention builds upon a specific pathophysiological model, the study results will provide increased insight in disease mechanisms, and may eventually foster similar studies in PPIS following other infections.

2. Hypotheses, aims and objectives

We hypothesise that a short-time outpatient-based rehabilitation program will be beneficial as compared to usual care in post-Covid syndrome

The aim of the present study is to assess the efficacy of a short-time outpatient-based rehabilitation program (the intervention) as compared to usual care (non-intervention) in post-Covid syndrome. The objectives are:

a) To determine the effect on physical functioning immediately after completion of the intervention. We hypothesises better physical functioning in the intervention group than in the non-intervention group.
b) To determine the effect on other post-Covid syndrome symptoms (such as fatigue, cognitive difficulties, dyspnoea, pain, sleeping problems) and quality of life (QoL) immediately after completion of the intervention. We hypothesise a beneficial effect of the intervention across all these domains.
c) To determine adverse effects of the intervention. We hypothesis no differences in the frequency nor seriousness of adverse effects in the intervention group as compared to the non-intervention group.
d) To determine the effect of the intervention on a long-term basis. We hypothesise that a beneficial effect of the intervention is sustained six months after its delivery.
e) To explore predictors of intervention effect, including differential effects across subgroups. We hypothesise that time since diagnosis of acute Covid-19 and hospitalization is negatively associated with intervention effect. Furthermore, we hypothesise equal effect of the intervention in the subgroup fulfilling diagnostic criteria for chronic fatigue syndrome/myalgic encephalomyelitis (CFS/ME).
f) To determine the cost-benefit and cost-effectiveness of the intervention. We hypothesise cost-benefit and cost-effectiveness to be better in the intervention as compared to the non-intervention group.

3. Project methodology

3.1. Project arrangements, method selection and analyses

Design, recruitment, inclusion/exclusion

The proposed study is a 2-arm pragmatic randomised controlled trial (RCT) in which 310 patients who suffer from post-Covid syndrome are randomised to either a short-time outpatient-based rehabilitation program (the intervention) or care as usual in a 1:1 ratio (Fig. 2). Assessments will take

place immediately before randomisation (T0), after intervention or care as usual (T1), and 12 months after T0 (T2). Of note, the timing of T1 will vary in the intervention group, due to the individualized therapy approach (cf. below); TI in the non-intervention group will be matched accordingly. Patients will be recruited from General Practitioners (GP's) as well as social media and self-referral to the involved institutions, and included based upon the criteria listed in Table 1.

Table 1. Criteria for inclusion and exclusion

Inclusion criteria	*Exclusion criteria*
Fulfils pragmatic diagnostic criteria of idiopathic post-Covid syndrome (Sandler 2021):<br>• Confirmed acute Covid-19 by a positive PCR or self-test for SARS-CoV-2.<br>• Persistent symptoms at least 3 months following acute Covid-19 without symptom-free interval.<br>• Functional disability to an extent that interrupts all or a majority of normal activities (such as work/school attendance, physical exercise, social activities, etc.)	Other chronic illnesses or demanding life situations that might explain persistent symptoms and disability<br><br>Sustained organ damage (lung, heart, brain) following acute, serious Covid-19
Age ≥ 16 years	Bedridden
Lives in one of the following Norwegian counties: Oslo, Viken, Innlandet, Vestfold og Telemark, Agder	Insufficient command of Norwegian language
Informed consent to participation	

Randomisation and blinding

Patients eligible for the present study are randomized to either intervention or care as usual in a 1:1 probability by a computer-based routine for block randomization; block size will vary randomly between 4 and 6. Allocation will be stratified by severity of illness during the acute stage of Covid-19, operationalized as (1) no admission to hospital, (2) admission to hospital. Allocation concealment will be ensured using sequentially numbered, opaque, sealed envelopes. It is not possible to blind for treatment due to the nature of the intervention. However, during endpoint-evaluation, the responsible researchers will be blinded for group allocation.

Intervention

The intervention consists of an individualized numbers of outpatient encounters (min. 2, max. 8) with medical doctors and physiotherapists at Kysthospitalet. The first meeting is always with a medical doctor. At least one of the encounters has to be face-to-face; the remaining encounters may be organized by video link for patients living far away. The encounters aim to foster a two-stage rehabilitation process:

- Stage 1: This first meeting is with a medical doctor. The intervention aims to eliminate serious pathology with a good anamnesis and clinical examination and to give patients` knowledge and understanding of their condition. The main focus is psychoeducation, based upon the theoretical framework of the Cognitive Activation Theory of Stress (CATS) and the Sustained Arousal-model of PPIS development. These models regard PPIS to be a product of functional brain alterations, which initially are an integrated part of the physiological acute sickness response,[1] but may become inappropriately sustained because of mechanisms such as unconscious expectancies/predictions, worrying tendencies and associative learning processes.[18,33,34,42] This sustainment causes persistent changes of autonomic nervous activity, hormonal activity and immunological responses, which in turn may explain patients' symptoms and occasional findings (such as high heart rate). Thus, the symptoms and functional disability of patients does not indicate a chronic infectious or autoimmuine disorders, and efforts are made to counteract patients' worries.
- Stage 2: The individual follow-up with physiotherapists starts with a repetition of the information given in stage 1, based on patients understanding of their condition and experienced symptoms.

The aim is to counteract patients` worries and subsequent symptom monitoring, and to reassure them that desired activities and graded exercise are both beneficial and safe. Over time, the overall aim is to increase activity levels and normalize activity tolerance by 'uncoupling' an unconscious association between activity and neurobiological stress response activation. The use of both cognitive talking therapy and behavioral modifications (included graded exercise) are important intervention components, and all the health care workers providing the intervention are educated in cognitive behavioral therapy (CBT). Stage 2 is highly individualized based upon the patients' symptom burden and personal preferences. A total of 120 patients` with post-Covid syndrome have received the intervention so far. The number of encounters needed as well as the interval between encounters is highly individual. It is considered important that the number of encounters is based on patients` individual needs, as a fixed number may delay recovery because of unnecessary treatments and result in overuse of health services.

The intervention share some resemblance with Cognitive Behavioral Therapy (CBT) for PPIS.[15,17] The rationale behind the intervention is explained in detail to the patients' family doctors, ensuring compliant follow-up in primary health care. The cost (in monetary units) will be calculated for both stages of the intervention.

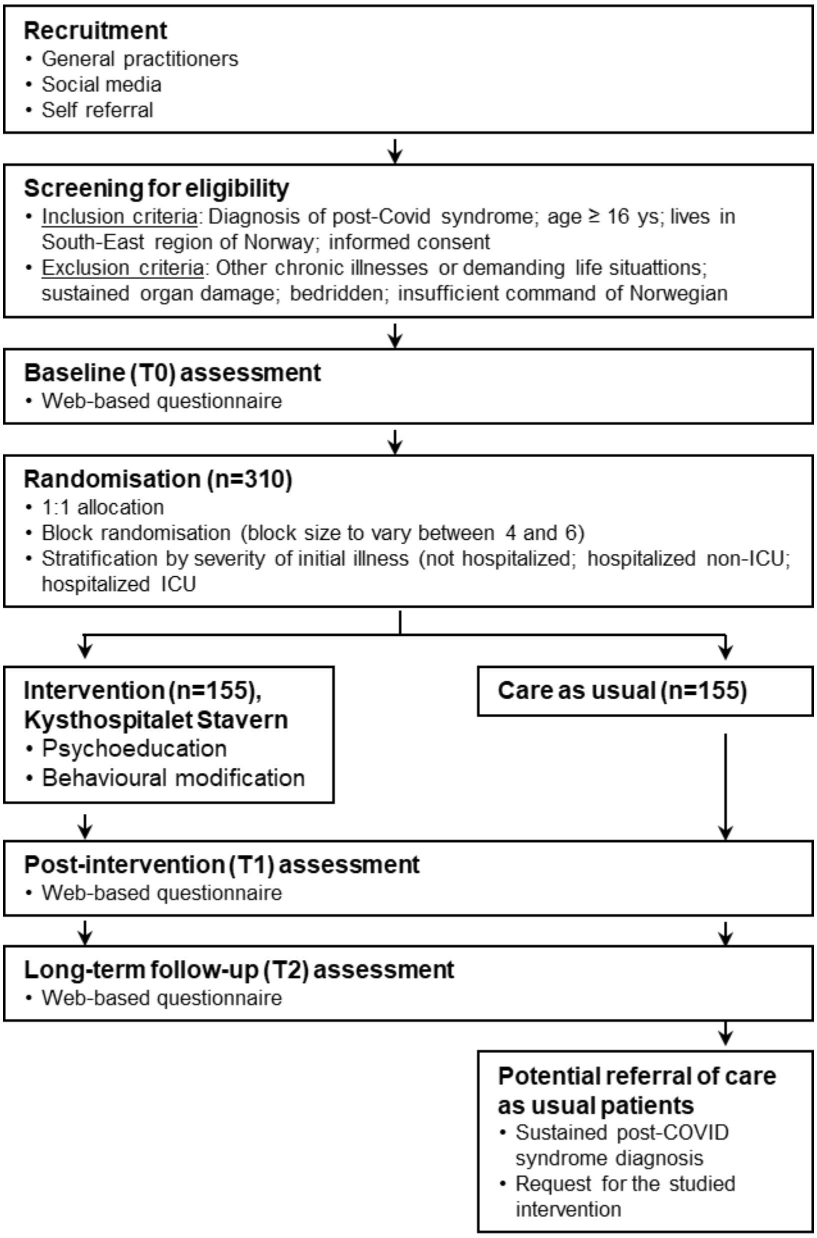

Figure 2. Overview of study design.

Effect monitoring
Effect monitoring is primarily based on patient reported outcome measures (PROM), applying a web-based questionnaire composed of several validated instruments. The questionnaire will be distributed to the participants at T0, T1 and T2. It will chart clinical symptoms as well as background, psychological and social variables, and will be used for subgrouping according to different case definitions of CFS/ME. The most important inventories are (cf. appendix 1 for a complete overview):

- *The 36-Item Short-Form Survey (SF-36)* comprises a total of 36 items on subjective mental, social and physical health, which are assigned to 8 dimensions. Scale ranging from 0 (poor quality of life) to 100 (best).[36,43] The SF-36 instrument was recently recommended as a useful tool to assess rehabilitation needs in post-Covid syndrome sufferers, and is generally recognized as a valid and reliable measurement for intervention trials.[20]
- *The Chalder Fatigue Questionnaire (CFQ)* charts subjective experience of physical and mental fatigue. In this study, the CFQ total linear score (i.e. the sum across all 11 items, each item scored on a zero to three Likert scale) will be applied. Furthermore, fatigue caseness is defined as a CFQ total dichotomous score of 4 or higher (each item scored 0-0-1-1).
- *The Hospital Anxiety and Depression Scale (HADS)* is a validated questionnaire for charting symptoms of depression and anxiety. It consists of 14 items rated zero to three on Likert scales, allowing computation of sub-scores for depression and anxiety symptoms. In the present study, these sub-scores as well as total sum score will be applied.
- *Adverse effects*: A self-invented questionnaire will address possible adverse effect related to the intervention.

In addition, employment status, usage of social security-systems (i.e. short and long-term sick leave), and usage of health care services (i.e. the number of GP and specialist contacts, hospitalisations, medications, usage of rehabilitation services, etc.) will be charted trough register linkage (cf. below) as well as questionnaires to the study participants.

In order to assess trajectories in potential improvement and explore mediation factors we will also administer the SF-36 inventory solely to the participants allocated to the intervention group after Stage 1 (ie., the first appointment with a medical doctor, cf. above).

Assessment of fidelity and dosage
During Stage 2, all the involved physiotherapists will receive a questionnaire assessing fidelity towards the intervention program after each patients encounter. Also, after completion of the rehabilitation process, each involved physiotherapist will be asked to quantify in broad terms the main interventions given (such as psychoeducation, guided exercises, etc.).

Unexpected findings of other health problems
The questionnaires may reveal unexpected findings of other health problems, such as serious depressive symptoms, substance abuse, etc. We will implement Standard Operating Procedures to ensure that such health problems are identified and that appropriate measures are instituted, such as immediate notifying of the patient's general practitioner/family doctor.

Linkage with health registries
In order to explore predictors of intervention effects (point 2.e), above), linkage with existing health registries will be considered, such as 'Norsk pasientregister', 'Mor-Barn-studien', 'Vaksine-registeret Sysvak', 'Reseptregisteret', 'Intensivregisteret' og 'Pandemiregisteret'. Also, previously recorded medical information from hospital and family doctor records will be exploited for the same purpose. A primary prediction analyses will use the following variables, based upon previous evidence from other studies of PPIS:[10,12,23]

- *Previous infectious diseases*: COVID-19 diagnosed by PCR-test (date, genetic variant), other infectious events one year prior to inclusion

- *Previous immunizations*: Vaccination against COVID-19 (date(s), type(s)), other vaccinations one year prior to inclusion.
- *Previous and current medical history*: Diagnoses of other chronic diseases, current medication
- *Severity of acute COVID-19*: Hospitalization (days), intensive care unit admission (days), respiratory support, cardiovascular support, neurological sequels, thromboembolic events, immunological and infectious markers during hospital stay (CRP, viral replication numbers).

Other possible predicting factors, such as genetic traits, will not be explored under the current protocol.

Population and sample size calculation
As of July 2021, a total of approximately 103,000 Covid-19 cases have been reported from the geographical areas (five Norwegian counties) from which patient are recruited to the present study. Assuming a 5 % case rate of post-Covid syndrome (cf. Figure 1) of whom 1/5 is eligible and willing to participate, a total of 1,300 patients could potentially be included.

The SF-36 subscale Physical Functioning (SF-36-PFS) will serve as the primary endpoint in the present study. A difference of 10 points is considered clinically significant.[43] Similarly, in a study of CFS/ME which shares similarities with post-Covid syndrome, the minimally clinically important difference of SF-36-PFS was reported to be 10.[4] The scatter of SF-36-PFS scores among post-Covid syndrome sufferers are unknown, but two large Norwegian surveys reported a Standard Deviation (SD) of 20 across all age groups.[8,14] If SD is set to be 25 in the population under study, and assuming 20 % drop-out rate, the study should aim to *include a total of 310 participants*. This yields a power of 90 % (α=0.05) to detect a small to medium effect size.

Statistical analyses
The ‘full analysis set’ (all randomized participants) will be used for intention-to-treat analyses of efficacy. Missing values will be imputed applying multiple imputation techniques. The ‘per protocol analysis set’ is defined as all patients in the ‘full analysis set’ that completed the treatment period without any of the following protocol deviations: Interruption of therapy; Lost to follow-up; Primary endpoint measurements missing; Diagnosed with another chronic disorder during the study period; Experiencing a severe illness or trauma during the study period; Commencing other treatment for post-Covid syndrome during the study period.

Variables will be reported with parametric or non-parametric descriptive statistics, eventually frequency tabulation, as appropriate. General linear models (ANCOVA) will be used for analyses of treatment effect; the baseline values of each efficacy endpoint will be included as covariates. For each statistical analysis, the net intervention effect (the mean change in the intervention group minus the mean change in the usual care group) will be calculated from the parameters of the fitted general linear model and reported with 95 % confidence interval. All statistical tests will be carried out two-sided. A p-value $\leq$ 0.05 is considered statistically significant. Safety data will be summarized descriptively through appropriate data tabulations and descriptive statistics. No interim analysis will be carried out. A statistical analysis plan will be developed.

3.2. Participants, organization and collaborations

Principal investigator and research group
The present project is hosted by the PAEDIA research group at Akershus University Hospital (Ahus), which has extensive experience in translational as well as clinical research on post-Covid syndrome and other PPIS, and a strong track record of result dissemination and implementation. The group has established a well-working infrastructure for patient flow and data acquisition, and a large collaborative network with national and international experts on various methodological aspects.

The PAEDIA research group is headed by Prof. Vegard Wyller, who is also the PI of the present project, and Chair of the COFFI collaborative (cf. below). Prof. Wyller is the most published and cited PPIS researcher in Norway. Other members of PAEDIA include two postdoctoral fellows, six PhD fellows, two master students, and three clinical consultants. The local research support staff

consists of one laboratory engineer, two research nurses and two research secretaries. An internal learning program for PhD fellows has been established, encompassing a) one-to-one supervision sessions; b) regular in-group discussions; c) invited lecturers; d) oral presentation training and practice; e) seminars on selected topics (statistics, ethics). A total of five PhD fellows have successfully graduated over the past four years.

Steering Committee, collaborating partners and networks

A Steering Committee consisting of Prof. Vegard Wyller (Head, Universituy of Oslo); Prof. Guri Rørtveit (University of Bergen); Prof. Signe Flottorp (Norwegian Institute of Public Health); Profs. Silje Endresen Reme and Henrik Børsting Jacobsen (Dept. of Psychology, University of Oslo) and Drs. Annika Jordbru, Andreas Horsdal, Gro Aasland, and Tom Farmen Nerli (Kysthospitalet Stavern) will oversee all stages of the project. The main collaborating partner is Kysthospitalet Stavern, where the intervention under study has already been established for other patient groups. In addition, the project will be intimately linked to an international consortium of researchers on post-infectious complications (Collaborative of Fatigue Following Infection, COFFI), encompassing highly reputed research groups from five countries (US, UK, Australia, The Netherlands and Norway), and with Prof. Wyller as the chair of the consortium (www.coffi-collaborative.com). A total of 15 post-infectious cohort studies (including 3 COVID-19 cohorts) have joined the collaborative so far, summing up to a total of about 6000 patients.

Important measures to ensure integration between all collaborators encompass a) Regular meetings (video link) to discuss scientific and strategic challenges; b) Hosting of seminars and workshops; and c) Face-to-face meetings.

The work tasks of a potential PhD fellow

A PhD fellow in the present project will be included in the team of researchers and managers responsible for participant investigations and data gathering. Thus, he will get first-hand knowledge of all parts of the investigational program described above. Furthermore, in close collaboration with his supervisors and the rest of the research team, he is expected to carry out data analyses, interpret the results and draft a total of four scientific papers.

Infrastructure

As the intervention under study has already been developed, the necessary research infrastructure is primarily related to a) administrative and coordinating tasks regarding patient flow, allocation, data management, etc; and b) patient recruitment from GPs. For the former, the project will exploit already established routines and personnel within the PAEDIA research group. For the latter, the project will benefit from using ‘Praksisnett’ – a collaborative network of GPs providing established routines and payment procedures for the recruitment of patients in clinical trials.

3.3. Plan for activities, visibility and dissemination

A PhD-dissertation can be completed within three years from project onset; the entire project period is estimated at a maximum of 10 years

A PhD-fellow focusing on research questions a) – c) will be able to complete a dissertation within approximately three years from project onset. However, extensive analyses related to research questions d) – f) are estimated to take longer time, and is also dependent on external funding of additional researchers (PhD and/or postdoctoral positions). Thus, the total project period is estimated at a maximum of 10 years.

Results will be disseminated widely; scientific dissemination is prioritized

Given the seriousness of post-Covid syndrome and the urgent need for scientific knowledge, rapid and continuous dissemination or project results will receive high attention. The target for dissemination is not restricted to the scientific community, but includes a wide range of health-care workers, politicians, policy makers, health care administrators, and the general public.

Several means of dissemination will be exploited, including scientific papers; popular science newspaper articles; giving interviews; meetings with politicians, public health providers and other stakeholders; social media. Dissemination to researchers and the scientific community includes publications in international, peer-reviewed, open access medical journals. The main results will be offered to journals of high impact. Negative findings will also be reported.

3.4. Plan for implementation

Based upon the research results, the Steering Committee will contribute actively to the establishment for clinical guidelines for post-Covid syndrome, in strong collaboration with health administrators, patients' organizations, and other stakeholders. A Working Group of should be established with the specific task of scrutinizing existing guidelines, revising them, and disseminating the revised guidelines.

4. User involvement

An advisory board of two user representatives is the most important user involvement

For the present project, an advisory board of users will formally established, consisting of a) One representative from the Consumer Advisory Committee at Ahus; and b) One representative from the organization Recovery Norway, consisting of individuals having recovered from PPIS and similar conditions. The advisory board will meet quarterly with the PI; important themes for discussions are results' interpretation and practical impact, ethical issues, considerations regarding further research, and dissemination/implementation strategies.

5. Ethical considerations

Ethics

Participation in the project is based upon informed consent, and thorough information will be provided orally as well as in writing to the participants. All data will be treated and stored without personal identifying information, and in accordance with national directives. Generally, investigational methods are neither harmful nor painful. Approval will be sought from the Regional Committees for Medical and Health Research Ethics. If the intervention is found to be beneficial, participants in the non-intervention group who suffer from a sustained post-Covid syndrome diagnosis will be offered a referral to Kysthospitalet for treatment after completion of the study (Fig. 2).

Gender perspectives

Severe COVID-19 appears to be more common in males than in females. post-infectious complications, however, such as PPIS, are more common among females than males, the ratio being about 3:1. Thus, such complications strongly impact on women's health, but have traditionally received low attention. In this project, possible gender differences will be scrutinized in the analyses.

6. References

1. Bennett BK, Goldstein D, Chen M, Davenport TA, Vollmer-Conna U, Scott EM, Hickie IB, Lloyd AR. Characterization of fatigue states in medicine and psychiatry by structured interview. Psychosom Med 2014; 76: 379-88.
2. Bestall JC, Paul EA, Garrod R, Garnham R, Jones PW, Wedzicha JA. Usefulness of the Medical Research Council (MRC) dyspnoea scale as a measure of disability in patients with chronic obstructive pulmonary disease. Thorax. 1999; 54: 581-586.
3. Boksem MAS, Tops M. Mental fatigue: cost and benefits. Brain Res Rev 2008; 59: 125-39.
4. Brigden A, Parslow RM, Gaunt D, Collin SM, Jones A, Crawley E. Defining the minimally linically important difference of the SF-36 physical function subscale for pediatric CFS/ME: triangulation using three different methods. Health Qual Life Outcome 2018; 16: 202.
5. Buchwald DS, Rea TD, Katon WJ, Russo JE, Ashley RL. Acute infectious mononucleosis: characteristics of patients who report failure to recover. Am J Med 2000; 109: 531-7.
6. Carvalho-Schneider C, Laurent E, Lemaignen A, Beaufils E, Bourbao-Tournois C, Laribi S, Flament T, Ferreira-Malden N, Bruyere F, Stefic K, Gaudy-Graffin C, Grammatico-Guillon L, Bernard L. Follow-up of adults with noncritical COVID-19 two months after symptom onset. Clin Microbiol Infect 2021; 27: 258-63.
7. Collin SM, Tilling K, Joinson C, Rimes KA, Pearson RM, Hughes RA, Sterne JAC, Crawley E. Maternal and childhood psychological factors predict chronic disabling fatigue at age 13 years. J Adolesc Health 2015; 56: 181-7.

8. Garratt AM, Stavem K. Measurement properties and normative data for the Norwegian SF-36: results from a general population survey. Health Qual Life Outcomes 2017; 15: 51.
9. Gherardi RK, Crépeaux G, Authier F-J. Myalgia and chronic fatigue syndrome following immunization: macrophagic myofasciities and animal studies support linkage to aluminium adjuvant persistency and diffusion in the immune system. Autoimmun Rev 2019; 18: 691-705.
10. Hanevik K, Wensaas KA, Rortveit G, Eide GE, Morch K, Langeland N. Irritable bowel syndrome and chronic fatigue 6 years after giardia infection: a controlled prospective cohort study. Clin Infect Dis 2014; 59: 1394-400.
11. Henningsen P, Gündel H, Kop WJ, Löwe B, Martin A, Rief, W, Rosmalen JGM, Schröder A, Fez-Cornelis C, van den Berg O. Persistent physical symptoms as perceptual dysregulation: A neuropsychobehavioural model and its clinical implications. Psychosom Med 2018; 80: 422-31.
12. Hickie I, Davenport T, Wakefield D, Vollmer-Conna U, Cameron B, Vernon SD, Reeves WC, Lloyd A. Post-infective and chronic fatigue syndromes precipitated by viral and non pathogens: prospective cohort study. BMJ 2006; 333: 575.
13. Institute of Medicine. Beyond myalgic encephalomyelitis/chronic fatigue syndrome. USA: The National Academies Press, 2015
14. Jacobsen EL, Bye A, Aass N, et al. Norwegian reference values for the Short-Form Health Survey 36: development over time. Qual Life Res 2018; 27: 1201-12.
15. Janse A, Work-Smeitink M, Bleijenberg G, Donders R, Knoop H. Efficacy of web-based cognitive behavioural therapy for chronic fatigue syndrome: randomized controlled trial. Br J Psychiatry 2018; 212: 112-8.
16. Jason LA, Cotler J, Islam MF, Sunnquist M, Katz BZ. Risks for developing ME/CFS in college students following infectious mononucleosis: a prospective cohort study. Clin Infect Dis 2020; Dec 25 [Online ahead of print].
17. Keijmel SP, Delsing CE, Bleijenberg G, van der Meer JWM, Donders RT, Leclercq M, Kampschreur LM, van den Berg M, Sprong T, Nabuurs-Franssen MH, Knoop H. Effectiveness of long-term doxycyline treatment and cognitive behavioural therapy on fatigue severity in patients with Q fever fatigue syndrome (Qure study): A randomized controlled trial. Clin Infect Dis 2017; 64: 998-1005.
18. Kube T, Rozenkrantz L, Rief E, Barsky A. Understanding persistent physical symptoms. Conceptual integration of psychological expectation models and predictrive processing accounts. Clin Psychol Rev 2020; 76: 101829.
19. Kube T, Rozenkrantz L, Rief W, Barsky A. Understanding persistent physical symptoms: Conceptual integration of psychological expectation models and predictive processing accounts. Clin Psychol Rev 2020: 101829.
20. Lemhöfer C, Gutenbrunner C, Schiller J, Loudovici-Krug D, Best N, Bökel A, Sturm C. Assessment of rehabilitation needs in patients after Covid-19: Development of the Covid-19-rehabilitation needs survey. J Rehab Med 2021; 53: jrm00183.
21. Montoya J, Holmes TH, Anderson KN, Maecker HT, Rosenberg-Hasson Y, Valencia IJ, et al. Cytokine signature associated with disease severity in chronic fatigue syndrome patients. Proc Natl Acad Sci USA 2017; 114: E7150-8.
22. Papadopoulos AS, Cleare AJ. Hypothalamic-pituitary-adrenal axis dysfunction in chronic fatigue syndrome. Nat Rev Endocrinol 2011; 8: 22-32.
23. Pedersen M, Asprusten TT, Godang K, Leegaard TM, Osnes LT, Skovlund E, Tjade T, Øie MG, Wyller VBB. Predictors of chronic fatigue in adolescents six months after acute Epstein-Barr virus infection: A prospective cohort study. Brain Behav Immun 2019; 75: 94-100.
24. Pilotto A, Cristillo V, Piccinelli SC, Zoppi N, Bonzi G, Sattin D, et al. COVID-19 severity impacts on long-term neuro-logical manifestation after hospitalisation. MedRxiv 2021: 2020.12.27.20248903.
25. Piraino B, Vollmer-Conna U, Lloyd AR. Genetic associations of fatigue and other symptom domains of the acute sickness response to infection. Brain Behav Immun 2012; 26: 552-8.
26. Sandler CX, Lloyd AR. Chronic fatigue syndrome: progress and possibilities. Med J Aust 2020; 212: 428-33.
27. Sandler CX, Wyller VBB, Moss-Morris R, Buchwald D, Crawley E, Hautvast J, Katz BZ, Knoop H, Little P, Taylor R, Wensaas K-A, Lloyd AR. Fatigue following COVID-19: Lessons learnt from post-infective fatigue syndrome. Open Forum Infectious Disease 2021; *in press.*
28. Seet RC, Quek AM, Lim EC. Post-infectious fatigue syndrome in dengue infection. J Clin Virol 2007; 38: 1-6.
29. Stavem K, Ghanima W, Olsen MK, Gilboe HM, Einvik G. Persistent symptoms 1.5 – 6 months after COVID-19 in non-hospitalised subjects: a population-based study. Thorax 2020; 76: 405-7.
30. Stavem K, Ghanima W, Olsen MK, Gilboe HM, Einvik G. Prevalence and determinants of fatigue after COVID-19 in non-hospitalized subjects: a population-based study. Int J Environ Res Public Health 2021; 18: 2030.
31. Sudre CH, Murray B, Varsavsky T, Graham MS, Penfold RS, Bowyer RC, et al. Attributes and predictors of Long-COVID: analysis of COVID cases and their symptoms collected by the Covid Symptoms Study App. MedRxiv 2020: 2020.10.19.20214494.
32. Sulheim D, Fagermoen E, Winger A, Andersen AM, Godang K, Müller F, Rowe PC, Saul JP, Skovlund E, Øie MG, Wyller VB. Disease mechanisms and clonidine treatment in adolescent chronic fatigue syndrome: a combined cross-sectional and randomized controlled trial. JAMA Pediatr 2014; 168: 351-60.
33. Ursin H, Eriksen HR. The cognitive activation theory of stress. Psychoneuroendocrinology 2004; 29: 567-92.
34. Van den Berg, Witthøft M, Petersen S, Brown RJ. Symptoms and the body: Taking the inferential leap. Neurosci Biobehav Rev 2017; 74: 185-203.
35. Van den Borst B, Peters JB, Brink M, Schoon Y, Bleeker-Rovers CP, Schers H, et al. Comprehensive health assessment three months after recovery from acute Covid-19. Clin Infect Dis 2020; Nov 21 [Online ahead of print].
36. Ware JE, Sherbourne CD. The MOS 36-itme short-form health survey (SF-36). I. Conceptual framework and item selection. Med Care 1992; 30: 473-83.
37. World Health Organization Coronavirus (COVID-19) Dashboard: https://covid19.who.int/. [Accessed April 10., 2021]
38. Wortinger LA, Endestad T, Melinder A, Øie MG, Sulheim D, Fagermoen E, Wyller VB. Emotional conflict processing in adolescent chronic fatigue syndrome: a pilot study using fMRI study. J Clin Experiment Neuropsychol 2017; 39: 355-68.
39. Wortinger LA, Endestad T, Sevenius A, Melinder A, Øie MG, Wyller VB. Aberrant resting-state functional connectivity in adolescent chronic fatigue syndrome. PLoS One 2016; 11: e0159351.
40. Wyller VB, Barbieri R, Saul P. Blood pressure variability and closed-loop baroreflex assessment in adolescent chronic fatigue syndrome during supine rest and orthostatic stress. Eur J Appl Physiol 2011; 111: 497-507.

41. Wyller VB, Due R, Saul JP, Amlie JP, Thaulow E. Usefulness of an abnormal cardiovascular response during low-grade head-up tilt-test for discriminating adolescents with chronic fatigue from healthy controls. Am J Cardiol 2007; 99: 997-1001.
42. Wyller VB, Eriksen HR, Malterud K. Can sustained arousal explain the chronic fatigue syndrome? Behav Brain Funct 2009; 5: 10.
43. Wyrwich KW, Thierney WM, Babu AN, et al. A comparison of clinically important differences in health-related quality of life for patients with chronic lung disease, asthma, or heart disease. Health Serv Res 2005; 40: 577–92

Appendix

Overview of inventories

Category	*Inventory*	*Characteristics*	*References*
**BACKGROUND/DEMOGRAPHICS**			
Household, socioeconomic level		Household members; parents work/education; chronic disease in family	
Smoking, alcohol, drugs		Alcoholic beverages, illicit drugs, smoking; 5-point Likert scale (never to each day)	
**SYMPTOMS AND FUNCTIONS**			
Fatigue	Chalder Fatigue Scale	11 items; 4-point Likert scale; each item scored 0-3; total sum score range 0 to 33.	Chalder T, Berelowitz G, Pawlikowska T, et al. J Psychosom Res 1993;37:147-53.
Chronic Fatigue Syndrome diagnosis and related symptoms	CDC symptom inventory for CFS	28 items addressing frequency of symptoms on 5-point Likert scale; each item scored 1-5 (never to each day/always).	Wagner D, et al. Popul Health Metr 2005;3:8
post-exertional malaise (PEM)	PEM items from DePaul Symptom Questionnaire	5 items addressing frequency of symptoms on 5 point Likert scale; each item scored 0-4 (never to each day/always). Average, then multiply with 25 to get a 100 point scoring scale.	Bedree H, et al. Fatigue 2019; 7: 166-79
Sleep disturbances	Karolinska sleep questionnaire (KSQ)	12 items addressing frequency of sleep related problems; 6-point Likert scale scored 1 – 6; lower score means *more* symptoms. Indexes for insomnia, awakening problems, and sleepiness	Akerstedt T, Ingre M, Broman JE, Kecklund G. Chronobiol Int 2008;25:333-48
Pain	Brief Pain Inventory (BPI)	4 items; 10 point Likert scale score 1 - 10 (no pain to worst pain possible)	Klepstad P, et al. J Pain Symptom Manage 2002;24:517-25.
Dyspnoea	Medical Research Council dyspnoea scale	1 item; 5 options (from “breathless during strenuous exercise” to “too breathless to leave the house”).	Bestall JC, et al. Thorax 1999; 54: 581-6.
Depression/Anxiety symptoms	Hospital Anxiety and Depression Symptoms (HADS)	14 items; 4 point Likert scale scored 0 – 3 (8 items with reversed scoring). Indexes for anxiety symptoms and depressive symptoms, also total sum score range 0 to 42.	Zigmond AS, Snaith RP. Acta Psychiatr Scand 1983;67:361-70.
Negative affect	Positive and Negative Affect Schedule (PANAS-SF)	5 items addressing negative affects (ashamed, anxious, nervous, hostile, upset); 5-point Likert scale scored 1-5. Total sum score range 5-25	Thompson ER. J of Cross-Cultural Psychology 2016; 38: 227-42.

Illness perception	Brief Illness Perception Questionnaire	8 items; 10 point Likert scale, scoring 1 – 10.	Broadbent E, et al. J Psychosom Res 2006;60:631-7
Work related self-efficacy	Return-to-work self-efficacy scale (RTWSE-11)	11 items; 6-point Likert scale, scored 1-6 (reverse scoring item 2 and 6). Total sum score range 11 to 66 (higher scores means high work-related self- efficacy).	Shaw WS, Reme SE, Linton SJ, et al. Scand J Work Environ Health 2011; 37: 109-19.
Quality of life	36-Item Short Form Survey (SF-36).	36 items, scored on Likert scales and recoded to achieve 100 point scales (higher score means better health); average score reported. Eight subdomains: Physical functioning; Role limitations due to physical health; Role limitations due to emotional problems; Energy/fatigue; Emotional well-being; Social functioning; Pain; General health	Ware JE, Sherbourne CD. Med Care 1992; 30: 473-83.
Health related quality of life	EuroQoL 5L (EQ-5D-5L)	5 items; 5 point Likert scale, scored 1 to 5. A summary index with a maximum score of 1 (best health status) can be derived from these five dimensions by conversion with a table of scores. In addition: 200 mm VAS scale for assessment on global health status. A score of 100 indicates best health status.	Herdman M, Gudex C, Lloyd A, Janssen MF, Kind P, Parkin D, et al. Quality of Life Research. 2011; 20: 1727-36.
Interoception	Body Vigilance Scale (BVS)	4 items; 11 point Likert scales 0 – 10. Score on item 3 should be devided by 10. Scores on item 4: average among 15 sensations. Total sum score range 0 to 40.	Schmidt NB, et al. J Consult Clin Psychol 1997; 65: 214-20.
Miscellaneous/hypotheses generating		a) 1 item addressing avoidance behavior: "To what degree do you avoid everything that may worsen your symptoms"?<br>b) 1 item addressing school/work absenteeism.	
**CONSTITUTIONAL**			
Neuroticism	NEO-FFI-30	6 items making up the neuroticism axis; 5-point Likert scale scored 0 – 4. Total sum score range 0 – 24.	Körner A, et al. Psychother Psychosom Med Psych. 2008; 58: 238-245
Worrying tendencies	Penn State Worry Questionnaire (PSWQ)	16 items; 5-point Likert scale scored 1 – 5 (reversed scoring of item1,3,8,10,11). Total sum score range 16 to 80	Pallesen, S, et al. Scandinavian Journal of Psychology 2006, 47, 281–291
Emotional awareness	Toronto Alexithymia Scale (TAS-20)	7 items making up the index of Difficult identifying feelings. 5-point Likert scale scored 1-5, total sum score range 7 to 49.	Bagby RM, et al.. J Psychosom Res 1994;38:33-40.

Loneliness	UCLA loneliness scale	20 items; 4-point Likert scale scored 1 – 4 (reverse scoring of item 1,5,6,9,10,15,16,19,20). Total sum score from 20 to 80.	Russell, D., et al. Journal of Personality and Social Psychology 1980, 39(3), 472–480
Self-efficacy	General Self-Efficacy Scale, short form	6 items; 4-point Likert scale scored 1 – 4. Total sum score from 6 to 24.	Romppel M, et al. Psychosoc Med 2013; 10: Doc01.
