# Supplementary material for: Brief Outpatient Rehabilitation Program for Post–COVID-19 Condition: A Randomized Clinical Trial
Source: JAMA Netw Open. 2024 Dec 19;7(12):e2450744. doi: 10.1001/jamanetworkopen.2024.50744 (PMC11659907; doi:10.1001/jamanetworkopen.2024.50744)

Statistical analyses plan

NCT05196451

February 23., 2022

1. DESIGN

Design overview

The proposed study is a 2-arm pragmatic randomised controlled trial (RCT) in which 310 patients who suffer from post-Covid syndrome are randomised to either a short-time outpatient-based rehabilitation program (the intervention) or care as usual in a 1:1 ratio (Fig. 1). Assessments will take place immediately before randomisation (T0), after intervention or care as usual (T1), and 12 months after T0 (T2). Of note, the timing of T1 will vary in the intervention group, due to the individualized therapy approach (cf. below); TI in the non-intervention group will be matched accordingly. Patients will be recruited from General Practitioners (GP's) as well as social media and self-referral to the involved institutions, and included based upon the criteria listed in Table 1.

Table 1. Criteria for inclusion and exclusion

Inclusion criteria	*Exclusion criteria*
Fulfils pragmatic diagnostic criteria of idiopathic post-Covid syndrome:<br>• Confirmed acute Covid-19 by a positive PCR or self-test for SARS-CoV-2.<br>• Persistent symptoms at least 3 months following acute Covid-19 without symptom-free interval.<br>• Functional disability to an extent that interrupts all or a majority of normal activities (such as work/school attendance, physical exercise, social activities, etc.)	Other chronic illnesses or demanding life situations that might explain persistent symptoms and disability<br><br>Sustained organ damage (lung, heart, brain) following acute, serious Covid-19
Age ≥ 16 years	Bedridden
Lives in one of the following Norwegian counties: Oslo, Viken, Innlandet, Vestfold og Telemark, Agder	Insufficient command of Norwegian language
Informed consent to participation	

Randomisation and blinding

Patients eligible for the present study are randomized to either intervention or care as usual in a 1:1 probability by a computer-based routine for block randomization; block size will vary randomly between 4 and 6. Allocation will be stratified by severity of illness during the acute stage of Covid-19, operationalized as (1) no admission to hospital, (2) admission to hospital. Allocation concealment will be ensured using sequentially numbered, opaque, sealed envelopes. It is not possible to blind for treatment due to the nature of the intervention. However, during endpoint-evaluation, the responsible researchers will be blinded for group allocation.

Effect monitoring

Effect monitoring is primarily based on patient reported outcome measures (PROM), applying a web-based questionnaire composed of several validated instruments. The questionnaire will be distributed to the participants at T0, T1 and T2. It will chart clinical symptoms as well as background, psychological and social variables, and will be used for subgrouping according to case definitions of post-COVID-19 syndrome and post-infective fatigue syndrome. The SF-36 subscale Physical Functioning (SF-36-PFS) score at T1 will serve as the **primary endpoint**.

Secondary endpoints include score of PROM inventories at T1 and T2, such as:

- *The Chalder Fatigue Questionnaire (CFQ)* charts subjective experience of physical and mental fatigue. In this study, the CFQ total linear score (i.e. the sum across all 11 items, each item scored on a zero to three Likert scale) will be applied. Furthermore, fatigue

caseness is defined as a CFQ total dichotomous score of 4 or higher (each item scored 0-0-1-1).

- *The Hospital Anxiety and Depression Scale (HADS)* is a validated questionnaire for charting symptoms of depression and anxiety. It consists of 14 items rated zero to three on Likert scales, allowing computation of sub-scores for depression and anxiety symptoms. In the present study, these sub-scores as well as total sum score will be applied.
- *Adverse effects*: A self-invented questionnaire will address possible adverse effect related to the intervention.

Cf. protocol with appendix for at detailed overview of inventories. In addition, employment status, usage of social security-systems (i.e. short and long-term sick leave), and usage of health care services (i.e. the number of GP and specialist contacts, hospitalisations, medications, usage of rehabilitation services, etc.) will be charted trough register linkage (cf. below) as well as questionnaires to the study participants.

Also, recovery from post-infective fatigue syndrome will serve as an endpoint. The appendix of the present statistical analysis plan provides an operationalization of post-infective fatigue syndrome according to the Fukuda-definition within the context of the present study.

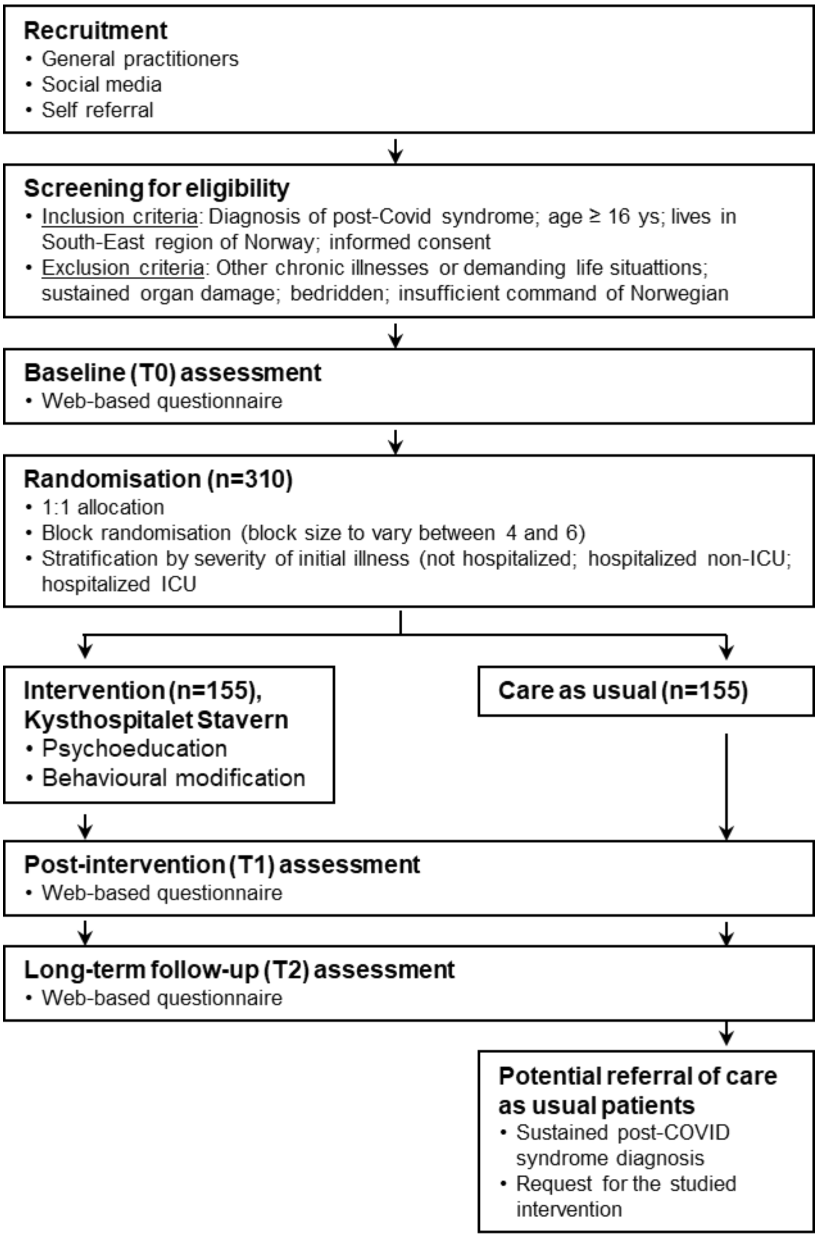

Figure 1. Overview of study design

2. POWER CONSIDERATIONS

For the SF-36-PFS score (primary endpoint), a difference of 10 points is considered clinically significant. Similarly, in a study of CFS/ME which shares similarities with post-Covid syndrome, the minimally clinically important difference of SF-36-PFS was reported to be 10. The scatter of SF-36-PFS scores among post-Covid syndrome sufferers are unknown, but two large Norwegian surveys reported a Standard Deviation (SD) of 20 across all age groups. If SD is set to be 25 in the population under study, and assuming 20 % drop-out rate, the study should aim to **include a total of 310 participants**. This yields a power of 90 % (α=0.05) to detect a small to medium effect size.

3. ANALYSIS SETS

Full analysis set

The ‘full analysis set’ is defined as all patients who were included and randomised This ‘full analysis set’ will be used for intention-to-treat analyses of efficacy. Missing values will be imputed based on the principle of multiple imputation, given a “missing-at-random”-pattern.

Per protocol analysis set

The ‘per protocol analysis set’ is defined as all patients in the ‘full analysis set’ that completed the treatment period and responded to the T1 questionnaire, without any of the following protocol deviations:

- Interruption of therapy.
- Lost to follow-up.
- Primary endpoint measurements missing.
- Diagnosed with another chronic disorder during the study period.
- Experiencing a severe illness or trauma during the study period.
- Commencing other treatment for post-COVID-19 condition during the study period.

Missing data will not be imputed in the per protocol analysis set.

4. STATISTICAL METHODS

General considerations

Continuous variables will be reported with parametric (mean/standard deviation) or non-parametric (median, quartiles) descriptive statistics, depending on the distribution. Ordinal/nominal variables will be reported as frequency tabulation. All statistical tests will be carried out two-sided. A p-value ≤ 0.05 is considered statistically significant.

Analyses of intervention effect

Intention-to-treat analyses (full analysis set) will be used to compare the group allocated to intervention with the group allocated to routine follow-up using general linear models (ANCOVA) for the primary endpoint. Analogous methodology within the generalized linear method group of statistical modeling will be used for secondary endpoints that are not linear. The baseline values of each efficacy endpoint as well as stratification variable will be included as covariates. The null hypothesis is no differences in efficacy variables between the two treatment allocation groups. For each statistical analysis, the net intervention effect (the mean change in the intervention group minus the mean change in the routine follow-up group) will be estimated from the parameters of the fitted model and reported with 95 % confidence interval. An identical methodological approach will be applied for per protocol analyses based upon the per protocol analysis set.

Safety data will be summarized descriptively through appropriate data tabulations and descriptive statistics. No interim analysis will be carried out.

Subgroup analyses

The outcome of the intervention in subgroups will be explored, based upon the full analysis set. Adherence to post-infective fatigue syndrome case definition at baseline (T0) will serve as the subgrouping variable. A differential outcome will be tested for efficacy variables at both time point, applying a generalized linear models including relevant interaction terms. Results of subgroup analyses will be presented if the interaction $p < 0.10$.

APPENDIX

Operationalization of post-infective fatigue syndrome (PIFS) caseness (modified Fukuda definition) at 6 months

1. The Fukuda definition, main criteria for PIFS (patients must adhere to all)

a) CFQ (Chalder Fatigue Questionnaire), total sum score at 6 months (dichotomous scoring 0-0-1-1 of single items)	≥ 4	Definition of fatigue caseness applied in several publications.
b) CFQ total sum score at baseline	≥ 4	Ensures persistence of fatigue from the acute infectious event.
c) SF-36; total score		Corresponds to the "fatigue severely affects daily activities" criterion, and to a chronic disease of "moderate" severity.
a) PEM (post-expertional malaise) frequency	≥ 2 for at least 1 of 5 items	From the DePaul symptom questionnaire. Corresponds to the "fatigue not alleviated by rest" criterion

2. The Fukuda definition, additional criteria for PIFS (criterion h) and i) are merged; patients must then adhere to at least 4 of 8)

a) "... experienced fatigue the day after an exertion"	≥ 3	Single item (5-point Likert scale, 3 indicates 1-2 times a week) from questionnaire
b) "… experienced headache"	≥ 3	Single item (5-point Likert scale, 3 indicates 1-2 times a week) from questionnaire
c) "… experienced sore throat"	≥ 3	Single item (5-point Likert scale, 3 indicates 1-2 times a week) from questionnaire
d) "… experienced tender cervical lymphatic nodes"	≥ 3	Single item (5-point Likert scale, 3 indicates 1-2 times a week) from questionnaire
e) "… experienced muscle pain"	≥ 3	Single item (5-point Likert scale, 3 indicates 1-2 times a week) from questionnaire
f) "… experienced multi-joint pain"	≥ 3	Single item (5-point Likert scale, 3 indicates 1-2 times a week) from questionnaire
g) "… experienced unrefreshing sleep"	≥ 3	Single item (5-point Likert scale, 3 indicates 1-2 times a week) from questionnaire
h) "… experienced concentration problems"	≥ 3	Single item (5-point Likert scale, 3 indicates 1-2 times a week) from questionnaire
i) "… experienced memory problems"	≥ 3	Single item (5-point Likert scale, 3 indicates 1-2 times a week) from questionnaire

3. Exclusion of other states that may explain fatigue (screening followed by individual evaluation)

3.1. SCREENING (INDIVIDUALS MUST ADHERE TO ALL IN ORDER TO REMAIN AS CASES; NON-ADHERENTS ARE SUBJECTED TO INDIVIDUAL EVALUATION, CF POINT 3.2)

a) HADS-A (Hospital Anxiety and Depression Scale, anxiety subscore)	≤ 10	Screening for anxiety. Score of 8-10 corresponds to "possible" anxiety caseness, 11-15 corresponds to "probable" anxiety caseness. A cut-off of 10 is reported to

		be optimal in a previous study of screening tools for psychiatric comorbidities in CFS/ME.
b) HADS-D (Hospital Anxiety and Depression Scale, depression subscore)	≤ 10	Score of 8-10 corresponds to “possible” depression caseness, 11-15 corresponds to “probable” depression caseness A cut-off of 10 is reported to be optimal in a previous study of screening tools for psychiatric comorbidities in CFS/ME
c) KSQ (Karolinska Sleep Questionnaire, total score)	≥ 2	Screening for primary sleep disorder
d) Other disorder/use of medications that may explain fatigue	No one	As reported in questionnaire, eg. psychiatric, cardiac, pulmonary, or rheumatic disease.
e) Substance abuse that may explain fatigue	No one	As reported in questionnaire

3.2. INDIVIDUAL EVALUATION OF POTENTIAL EXCLUSIONS (INDIVIDUALS **EXCLUDED** AS CASES MUST ADHERE TO ALL)*

a) Is it likely that the disorder/aberration is causally related to persistent fatigue?	Yes

*Individual evaluation is performed independently by two researchers, using all available information such as recorded data in the present project as well as patients’ hospital and GP records. If disagreement about classification, cases are discussed with the principal investigator of the project until consensus is reached. A sensitivity analysis will be carried out excluding all cases with uncertain classification.

Algorithm for caseness assessment

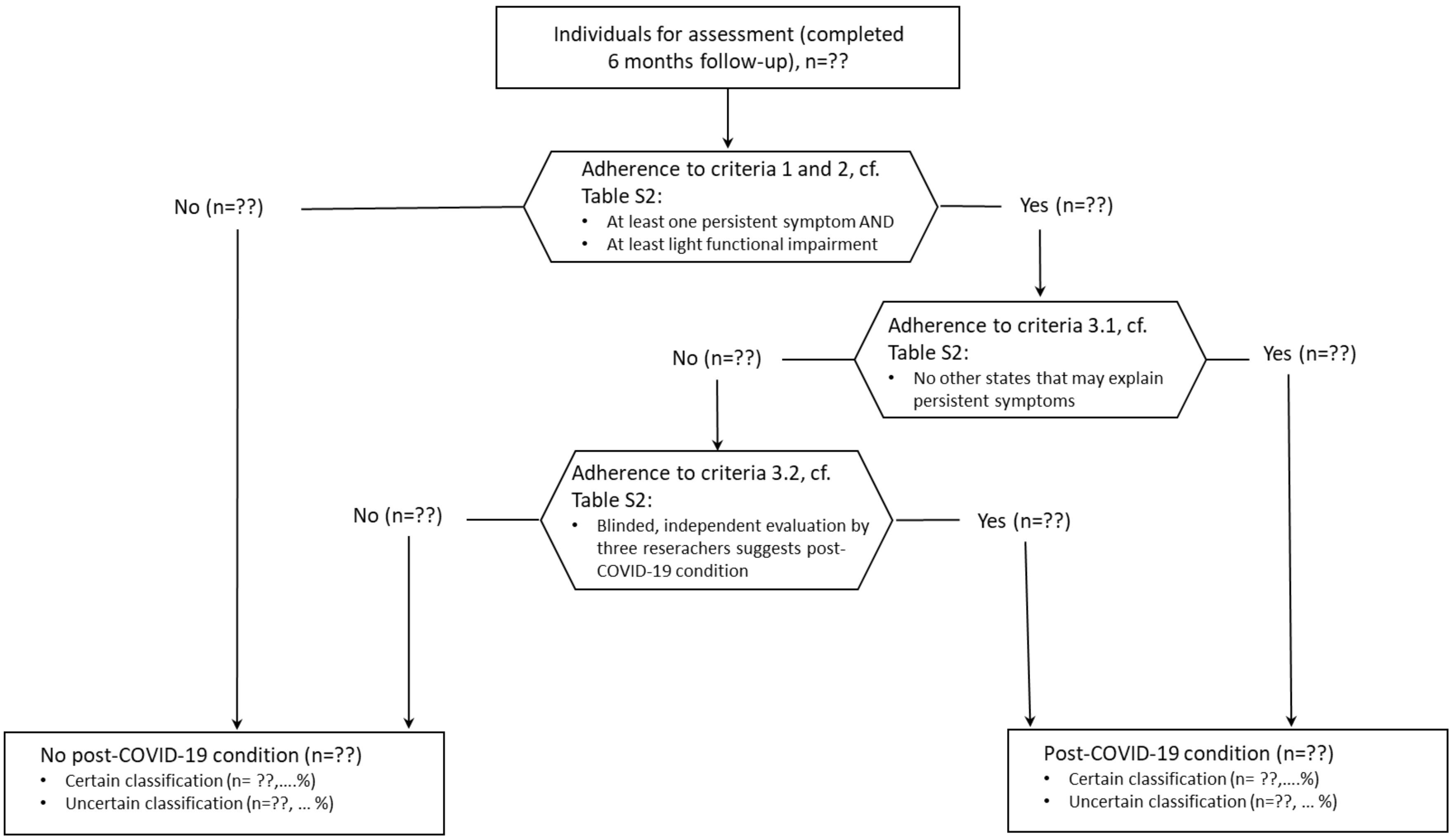

Supplement: Supplement 2. — Statistical Analysis Plan [file jamanetwopen-e2450744-s002.pdf]
