# Supplementary material for: Brief Outpatient Rehabilitation Program for Post–COVID-19 Condition: A Randomized Clinical Trial
Source: JAMA Netw Open. 2024 Dec 19;7(12):e2450744. doi: 10.1001/jamanetworkopen.2024.50744 (PMC11659907; doi:10.1001/jamanetworkopen.2024.50744)
Supplement: Supplement 3. — eTable 1. Content Topics and Fidelity to Content Topics in All Consultations (n=516) at Stage Two of the Rehabilitation Program eTable 2. Symptom Prevalence at Baseline eTable 3. Attritions eTable 4. Lost to Follow-Up eTable 5. Protocol Deviations eTable 6. Sensitivity Analysis 1 eTable 7. Sensitivity Analysis 2 eTable 8. Participants Meeting Recovery Threshold of Self-Reported Physical Function, per-Protocol Data eTable 9. Subgroup Analyses According to PIFS Caseness at Inclusion eTable 10. Subgroup Analyses According to PEM Score at Inclusion eTable 11. Adverse Events eTable 12. Decrease in SF-36 Physical Function Subscore From Baseline to T1 in the Intervention Group, Qualitative Evaluation eTable 13. Increase in Post Exertional Malaise With DePaul Symptom Questionnaire Addressing Frequency of Symptoms From Baseline to T1 in the Intervention Group, Qualitative Evaluation eFigure 1. Adherence to the Modified Fukuda-Definition of PIFS Caseness eFigure 2. Correlation (Spearman’s Rho) Heatmap of Symptom-End Points and Selected Symptoms eFigure 3. Physical Function Score (0-100) and Trajectories of Participants Who Attended Any Follow-Up Timepoint eReferences [file jamanetwopen-e2450744-s003.pdf]

Supplementary Online Content

Nerli TF, Selvakumar J, Cvejic E, et al. Brief outpatient rehabilitation program for post–COVID-19 condition: a randomized clinical trial. *JAMA Netw Open*. 2024;7(12):e2450744. doi:10.1001/jamanetworkopen.2024.50744

eTable 1. Content Topics and Fidelity to Content Topics in All Consultations (n = 516) at Stage Two of the Rehabilitation Program

eTable 2. Symptom Prevalence at Baseline

eTable 3. Attritions

eTable 4. Lost to Follow-Up

eTable 5. Protocol Deviations

eTable 6. Sensitivity Analysis 1

eTable 7. Sensitivity Analysis 2

eTable 8. Participants Meeting Recovery Threshold of Self-Reported Physical Function, per-Protocol Data

eTable 9. Subgroup Analyses According to PIFS Caseness at Inclusion

eTable 10. Subgroup Analyses According to PEM Score at Inclusion

eTable 11. Adverse Events

eTable 12. Decrease in SF-36 Physical Function Subscore From Baseline to T1 in the Intervention Group, Qualitative Evaluation

eTable 13. Increase in Post Exertional Malaise With DePaul Symptom Questionnaire Addressing Frequency of Symptoms From Baseline to T1 in the Intervention Group, Qualitative Evaluation

eFigure 1. Adherence to the Modified Fukuda-Definition of PIFS Caseness

eFigure 2. Correlation (Spearman's Rho) Heatmap of Symptom-End Points and Selected Symptoms

eFigure 3. Physical Function Score (0-100) and Trajectories of Participants Who Attended Any Follow-Up Timepoint

eReferences

This supplementary material has been provided by the authors to give readers additional information about their work.

© 2024 Nerli TF et al. *JAMA Network Open.*

eTable 1. Content topics and fidelity to content topics in all consultations (n = 516) at stage two of the rehabilitation program.

Topic	N consultations addressing topic	Proportion of consultations addressing this topic (N/n)	Mean fidelity scores[a] (CI)	*Brief description of content*
**Update and mood check**	510	0.99	4.73(4.65, 4.80)	*In-session structure.*<br>*Always give the patients time to tell their story, new developments, good or bad experiences since the last session. Fostering therapeutic alliance based on trust and mutual respect.*
**Repetition of CATS**[1]	83	0.16	4.47 (4.21, 4.73)	*Cognitive reassurance*[2] *and stimulus expectancies.*[3]<br>*Providing explanation and education. CATS is given in summary by a drawing on a blackboard or by power point in video-link.*
**Explanation of symptoms within CATS**	222	0.43	4.36 (4.25, 4.47)	*Cognitive reassurance and stimulus expectancies.*<br>*Explaining how an abnormal perpetuation of the acute sickness response can give a wide range of bodily symptoms because of changes in autonomic nervous activity, hormonal activity and immunological activity. Experienced symptoms are viewed as natural after infection, the symptoms will vary but the symptoms are independent of activity and not related to organ damage.*
**Symptom monitoring as a perpetuating factor**	221	0.43	4.54 (4.43, 4.65)	*Stimulus expectancies.*<br>*Explaining that symptoms are expected and will vary. Progress is not monitored in more or less symptoms, but more or less actions. Thus, the patients are asked if it is possible to let go of constant monitoring and evaluation of their symptoms since decrease in symptoms itself is not a goal of the treatment.*
**Associative learning regarding activities and symptoms as a perpetuating factor**	192	0.37	4.53 (4.41, 4.66)	*Stimulus expectancies.*<br>*An increase in symptoms does not necessarily mean that patients have done something wrong.*
**The body`s ability to adapt**	389	0.75	4.66 (4.58, 4.75)	*Stimulus expectancies.*

© 2024 Nerli TF et al. *JAMA Network Open.*

				Aiming to foster a belief that patients` bodies have the potential for recovering and that the disorder is temporary and amendable.
**What is energy?**	68	0.13	4.26 (4.04, 4.48)	*Stimulus expectancies.*<br>*Energy can`t be viewed as a battery. The feeling of more or less energy is related to CATS and a brief or sustained stress-response. Thus, the feeling of energy will vary, but the feeling of more or less energy is not a trusted tool for deciding to pursue a pleasurable activity or not.*
**Discomfort is sometimes necessary**	289	0.56	4.34 (4.24, 4.43)	*Stimulus expectancies.*<br>*Every stimulus is followed by a bodily response, sometimes that response might be unpleasant. Still, an increase in symptoms does not necessarily mean that patients have done something wrong. Continuity in exploring activities is still viewed as important.*
**Avoidance behavior**	179	0.35	4.28 (4.16, 4.41)	*Stimulus expectancies.*<br>*Avoidance has the potential for a less flexible approach towards activities and work. Avoidance is a perpetuating factor. "It is not what you avoid that makes you better, it is what you do."*
**Stimulus and load are necessary**	445	0.86	4.63 (4.55, 4.71)	*Stimulus expectancies.*<br>*Recovery would require an active pursuit of physical and mental tasks.*
**Exploring activities as something positive, shared decision making on what to explore**	314	0.61	4.55 (4.47, 4.64)	*Response outcome expectancies.*[3]<br>*Getting to know the patients, their interests and values is a priority. What activities are they missing at the moment, and what do they want to pursuit moving forward? Is the choice of activity/activities suitable, feasible and enjoyable over time?*
**Work/education as something positive**	331	0.64	4.61 (4.52, 4.71)	*Response outcome expectancies.*<br>*Work can promote better health under the right conditions. Are we in a situation where work can be an activity to explore?*
**Practical exposure therapy, exploring activities**	17	0.03	4.94 (4.56, 5.33)	*Response outcome expectancies.*<br>*Exploring activities together with the patients for reassurance and coping.*

© 2024 Nerli TF et al. *JAMA Network Open.*

**Theoretical exposure therapy, exploring activities**	182	0.35	4.56 (4.45, 4.67)	*Response outcome expectancies.*<br>*Discussing how favorable experiences would induce coping.*
**Structured advice for graded exercise**	89	0.17	4.48 (4.31, 4.66)	*Some patients want concrete advice on training, content, duration, intensity and progression. This is only provided if the patients themselves want to explore training in a more structured manner.*
**Accept and understanding for the patient's situation and symptoms**	458	0.89	4.62 (4.53, 4.71)	*In-session structure.*<br>*Always give the patients time to tell their story, new developments, good or bad experiences since the last session. Understanding and empathy is important and always validating symptoms as real experiences since they are real.*
**Socratic dialogue**[4]	332	0.64	N/A[b]	*In-session structure.*<br>*Facilitating for an open, respectful and safe dialogue. Help patients to evaluate their thinking and conclusions rather than just giving facts. Enable patients to draw conclusions themselves.*
**Homework,**[5] **shared decision making**	331	0.64	4.43 (4.33, 4.52)	*In-session structure and response outcome expectancies.*<br>*In-session procedure for discussing the homework. As the treatment is the patients` project they are the ones who decides homework and gives feedback on its benefits the next session. Adherence is important, we always ask if the homework is suitable, feasible and enjoyable.*<br>*The patients are encouraged, through homework, to actively explore new activities with an understanding that it is both safe and necessary for improvement. Over time, it is a clear aim that favorable experiences will induce coping.*
**Both patients and therapists` summarizes the session**	448	0.87	4.52 (4.44, 4.61)	*In-session structure.*<br>*Therapists listen, try to understand, then ask for permission to give information, possibly giving facts and then summarizes each content that is brought up during the session. Also summarizing at the end to ensure that there is a shared understanding between the patient and the therapist.*<br>*The patients are also always asked for summarizing content discussed during the session and at the end for the purpose of shared understanding.*

© 2024 Nerli TF et al. *JAMA Network Open.*

Abbrevations: CATS, the Cognitive activation theory of stress; CI, 95 % confidence interval; N/A, not applicable

[a]Immediately following the consultation, therapists reported fidelity on a Likert scale ranging from 0 to 6, where a higher score indicates better fidelity.

[b]Fidelity was not assessed for ‘socratic dialogue’.

© 2024 Nerli TF et al. *JAMA Network Open.*

eTable 2. Symptom prevalence at baseline (n = 314)[a]

Symptoms[b]	Prevalence, No.(%)	Category(-ies)
Fatigue	299 (96.5)	Fatigue and PEM
**Fatigue the day after exertion**	293 (94.5)	Fatigue and PEM
Unrefreshing sleep	283 (91.3)	Fatigue and PEM
Concentration problems	278 (89.7)	Cognitive
**'Empty batteries' after light activities**	276 (89)	Fatigue and PEM
**Tired 'in the head' after minimal exertions**	271 (87.4)	Fatigue and PEM
**Extraordinary fatigue after physical activity**	269 (86.8)	Fatigue and PEM
Memory problems	261 (84.2)	Cognitive
**Lack of muscle strength even after resting**	235 (75.8)	Fatigue and PEM
Dizziness	224 (72.3)	Autonomic
Difficulties making decisions	216 (69.7)	Cognitive
Headache	213 (68.7)	General infectious
Muscle pain	196 (63.2)	General infectious
Sensitive to sounds	189 (61)	Other
**Muscle soreness after normal daily activities**	175 (56.5)	Fatigue and PEM
Shortness of breath	167 (53.9)	Respiratory
Multi-join pain	166 (53.5)	General infectious
Palpitations	163 (52.6)	Cardiac / Autonomic
Tinnitus	154 (49.7)	ENT
Abdominal bloating	151 (48.7)	Abdominal
**Feel alternately hot and cold**	142 (45.8)	Autonomic
Cough	132 (42.6)	ENT / Respiratory
Abnormal stools	132 (42.6)	Abdominal
Nausea	128 (41.3)	Abdominal

© 2024 Nerli TF et al. *JAMA Network Open.*

Chest pain	121 (39)	Cardiac
Numb extremities	114 (36.8)	Other
Runny nose	111 (35.8)	ENT
Feeling of fever	109 (35.2)	General infectious
Abdominal pain	108 (34.8)	Abdominal
Sensitive to light	107 (34.5)	Other
Confused / disoriented	105 (33.9)	Cognitive
**Problems with sense of smell**	94 (30.3)	ENT
**Problems with sense of taste**	87 (28.1)	ENT
Pale and cold hands	86 (27.7)	Autonomic
Sore throat	82 (26.5)	ENT
Tender lymphatic nodes	42 (13.5)	General infectious

Abbrevations: ENT, Ear-nose-throat; PEM, Post-exertional malaise
[a] Four participants did not answer baseline questionnair
[b]With the exception of 'fatigue', all symptoms were self-reported on a Likert scale 1-5, with 1 corresponding to 'Never' and 5 to 'Constantly'. The prevalence presented is for reporting a value of three or higher.
[c]From the Chalder Fatique Questionnaire[6]; prevalence reported is for a total score of 4 or higher, using the bimodal scoring method.

© 2024 Nerli TF et al. *JAMA Network Open.*

eTable 3. Attritions

Characteristics of individuals invited to the study, and their association to declining the invitation to participate. Logistic regression.

Variable	Invited (n=464)	Declined (n=150)	Accepted/included (n=314)	Odds ratio	p-value
**Age - mean (95% CI)**	42.5 (41.3, 43.7)	42 (39.5, 44.5)	42.8 (41.4, 44.1)	1.00 (0.98, 1.01)	0.55
**Female sex - No. (%)**	332 (71.6)	107 (71.3)	225 (71.7)	1.02 (0.66, 1.56)	0.94
**Self-referred[a] - No. (%)**	103 (22.2)	11 (7.3)	92 (29.3)	0.19 (0.09, 0.36)	<0.001

Abbrevations: CI; confidence interval.
[a]Participants were recruited through self-referral or physician-referral

© 2024 Nerli TF et al. *JAMA Network Open.*

eTable 4. Lost to follow-up.

Association of baseline characteristics and symptoms being lost to follow-up. Logistic regression, per protocol data.

	Baseline characteristics		OR of being lost to follow-up	
**Variable**	All (n=314)[a]	Lost to follow-up at T1(N=83)[b]	Odds ratio (95% CI)	p-value
**Any comorbidity - No. (%)**	123 (39.7)	34 (43)	0.83 (0.495, 1.4)	0.48
**Female sex - No. (%)**	225 (71.7)	56 (67.5)	0.792 (0.457, 1.4)	0.41
**Allocated to intervention group - No.(%)**	157 (50)	32 (38.6)	0.492 (0.288, 0.827)	0.008
**Age**	42.8 (41.4, 44.1)	39 (36.1, 41.8)	1.04 (1.01, 1.06)	0.001
SF-36 subscores				
Physical function[c]	63.2 (60.8, 65.6)	63.3 (58.4, 68.2)	1 (0.988, 1.01)	0.95
Role limitations due to due to physical health problems[d]	7.58 (5.29, 9.87)	6.96 (2.58, 11.3)	1 (0.99, 1.02)	0.76
Bodily pain[e]	48.8 (47.7, 50)	49.3 (46.8, 51.8)	0.994 (0.969, 1.02)	0.64
General health[f]	44.4 (41.9, 46.8)	40.4 (35.6, 45.3)	1.01 (0.999, 1.02)	0.07
Vitality[g]	22.2 (20.6, 23.9)	22.9 (19.8, 26)	0.996 (0.979, 1.01)	0.62
Social function[h]	40.6 (37.7, 43.4)	42.9 (37.7, 48.1)	0.995 (0.985, 1.01)	0.35
Role limitiations due to emotional problems[i]	55.7 (50.7, 60.7)	58.6 (49, 68.3)	0.998 (0.992, 1)	0.49
Mental health[j]	63.2 (61.2, 65.1)	60.5 (56.7, 64.3)	1.01 (0.997, 1.03)	0.12
Symptoms				
Fatigue[k]	25.1 (24.6, 25.7)	24.6 (23.5, 25.7)	1.03 (0.976, 1.08)	0.29
Post-exertional malaise[l]	65.5 (62.8, 68.1)	64.6 (59, 70.1)	1 (0.991, 1.01)	0.70
Breathlessness[m]	1.1 (1.0, 1.2)	1.08 (0.87, 1.28)	1.03 (0.78, 1.38)	0.81
**Cognitive symptoms[n]**	3.3 (3.2, 3.4)	3.3 (3.1, 3.5)	1.03 (0.79, 1.34)	0.82
**Loss of taste[o]**	2.08 (1.91, 2.26)	2.15 (1.81, 2.49)	0.962 (0.82, 1.13)	0.64
Loss of smell[p]	2.01 (1.84, 2.18)	1.97 (1.65, 2.3)	1.02 (0.866, 1.22)	0.80
Sleep quality[q]	3 (2.91, 3.08)	3 (2.83, 3.16)	1 (0.714, 1.41)	0.99

© 2024 Nerli TF et al. *JAMA Network Open.*

Anxiety symptoms[r]	7.67 (7.18, 8.16)	8.43 (7.42, 9.44)	0.949 (0.896, 1.01)	0.08
Depressive symptoms[s]	6.92 (6.47, 7.37)	6.8 (6, 7.6)	1.01 (0.948, 1.08)	0.75

Abbrevations: Abbreviations: OR, Odds Ratio SF-36, Short Form Health Survey; CI, confidence interval; T1, first follow-up, after completion of the intervention (approximately 6 months after inclusion.

[a]Four individuals were lost to follow-up before T0, and thus the values presented in the column are that of 310 individuals (except for the allocation variable).

[b]Four out of 83 individuals were lost to follow-up before T0

[c]Based upon 10 single items from the Short Form Health Survey[7]; total range 0-100 where higher scores imply better physical function.

[d]Based upon 4 single items from the Short Form Health Survey[7]; total range 0-100 where higher scores imply fewer limitations due to physical problems.

[e]Based upon 2 single items from the Short Form Health Survey[7]; total range 0-100 where higher scores imply less pain.

[f]Based upon 5 single items from the Short Form Health Survey[7]; total range 0-100 where higher scores imply better general health.

[g]Based upon 4 single items from the Short Form Health Survey[7]; total range 0-100 where higher scores imply better vitality.

[h]Based upon 2 single items from the Short Form Health Survey[7]; total range 0-100 where higher scores imply better social functioning.

[i]Based upon 3 single items from the Short Form Health Survey[7]; total range 0-100 where higher scores imply fewer limitations due to emotional problems.

[j]Based upon 5 single items from the Short Form Health Survey[7]; total range 0-100 where higher scores imply better mental health.

[k]Based upon 11 single items from the Chalder Fatigue Scale[6]; total range 0-33 where higher scores imply more fatigue.

[l]Based upon 5 single items from the DePaul Symptom Questionnaire[8] addressing frequency of symptoms; total range 0-100 where higher scores imply more post-exertional malaise.

[m]Based upon 1 single item from the Medical Research Council Dyspnoea Scale[9]; total range 0-4 where higher scores imply more dyspnea.

[n]Based upon the average of 4 single items addressing memory, concentration, confusion and ability to take decisions used in a previous Covid-19 cohort study[10]; total range 1-5 where higher scores imply more cognitive difficulties.

[o]Based upon the average of 2 single items addressing smell and taste abnormalities, respectively, used in a previous Covid-19 cohort study[10]; total range 1-5 where higher scores mean fewer smell/taste abnormalities.

[p]Based upon the average of 2 single items addressing smell and taste abnormalities, respectively, used in a previous Covid-19 cohort study[10]; total range 1-5 where higher scores mean fewer smell/taste abnormalities.

[q]Based upon 12 single items from the Karolinska Sleep Questionnaire[11] addressing frequency of sleep related problems; total range 12-72 where higher scores imply better sleep.

[r]Based upon 7 single items from the Hospital Anxiety and Depression Scale anxiety subscale[12]; total range 0-21 where higher scores imply more symptoms related to anxiety.

[s]Based upon 7 single items from the Hospital Anxiety and Depression Scale depression subscale[12]; total range 0-21 where higher scores imply more symptoms related to depression.

© 2024 Nerli TF et al. *JAMA Network Open.*

eTable 5. Protocol deviations

**Protocol deviations[a] reported during the intervention period (T0 - T1)**		
Category	**Care as usual**	**Intervention**
Primary endpoint missing - no	51	32
Lost to follow up included study drop out - no.	38	23
Interruption of therapy - no.	N/A	3
Diagnosed with another chronic disorder - no.	1	3
Experiencing severe illness/trauma - no.	0	1
Receiving treatment at Kysthospitalet while in group care as usual - no	1	N/A
Commencing inpatient rehabilitation for Long COVID - no.	N/A	5
**Totals – no**	**91**	**67**

Abbrevations: T0, inclusion timepoint; T1, first follow-up, after completion of the intervention; N/A, not applicable; Kysthospitalet, Division of Physical Medicine and Rehabilitation, Vestfold Hospital Trust, Tønsberg, Norway.
[a]One individual may have more than one protocol deviation

© 2024 Nerli TF et al. *JAMA Network Open.*

eTable 6. Sensitivity analysis 1.

**Outcome of the intervention. Per-protocol analyses[a]**				
		**T1 (n=224)**		**T2 (n=224)**
Primary endpoint, SF-36 subscore				
Physical function[b]	Care as usual - mean	68.4		69.4
	Intervention - mean	80.4		80.6
	Difference (95 % CI)	12.0 (8.0 to 15.9)		11.2 (6.6 to 15.8)
	p-value	<0.001		<0.001
	Cohen's *d*	0.58		0.54
Secondary endpoints, SF-36 subscores				
Role limitations due to physical problems[c]	Care as usual - mean	15.9		19.3
	Intervention - mean	30.7		40.6
	Difference (95 % CI)	14.8 (5.5 to 24.1)		21.4 (10.7 to 32.1)
	p-value	0.002		<0.001
	Cohen's *d*	0.39		0.52
Bodily pain[d]	Care as usual - mean	51.2		50.5
	Intervention - mean	52.5		53.8
	Difference (95 % CI)	1.3 (-1.2 to 3.8)		3.4 (0.7 to 6.0)
	p-value	0.32		0.01
	Cohen's *d*	0.13		0.36
General health[e]	Care as usual - mean	42.7		42.7
	Intervention - mean	55.2		55.0

© 2024 Nerli TF et al. *JAMA Network Open.*

	Difference (95 % CI)	12.5 (7.7 to 17.3)		12.3 (6.5 to 18.1)
	p-value	<0.001		<0.001
	Cohen's *d*	0.55		0.49
Vitality[f]	Care as usual - mean	29.3		26.9
	Intervention - mean	40.7		39.3
	Difference (95 % CI)	11.4 (6.5 to 16.2)		12.4 (7.3 to 17.5)
	p-value	<0.001		<0.001
	Cohen's *d*	0.54		0.60
Social functioning[g]	Care as usual - mean	49.0		48.5
	Intervention - mean	65.8		68.2
	Difference (95 % CI)	16.7 (10.7 to 22.7)		19.7 (13.0 to 26.3)
	p-value	<0.001		<0.001
	Cohen's *d*	0.63		0.71
Role limitations due to emotional problems[h]	Care as usual - mean	55.9		51.6
	Intervention - mean	62.5		71.2
	Difference (95 % CI)	6.6 (-4.5 to 17.7)		19.6 (8.6 to 30.5)
	p-value	0.24		0.001
	Cohen's *d*	0.15		0.44
Mental health[i]	Care as usual - mean	67.0		66.5
	Intervention - mean	73.6		74.1
	Difference (95 % CI)	6.6 (3.2 to 10.0)		7.6 (4.2 to 11.0)
	p-value	<0.001		<0.001
	Cohen's *d*	0.40		0.48
Secondary endpoint, working abilities				

© 2024 Nerli TF et al. *JAMA Network Open.*

Return to work self-efficacy[j]	Care as usual - mean	3.1		3.2
	Intervention - mean	3.8		3.9
	Difference (95 % CI)	0.64 (0.37 to 0.91)		0.70 (0.38 to 1.0)
	p-value	<0.001		<0.001
	Cohen's *d*	0.50		0.54
Secondary endpoints, symptoms				
Fatigue[k]	Care as usual - mean	21.5		20.9
	Intervention - mean	18.2		17.8
	Difference (95 % CI)	-3.3 (-5.1 to -1.6)		-3.1 (-4.8 to -1.4)
	p-value	<0.001		0.001
	Cohen's *d*	0.45		0.44
Post-exertional malaise[l]	Care as usual - mean	55.0		54.8
	Intervention - mean	38.6		39.3
	Difference (95 % CI)	-16.4 (-22.7 to -10.0)		-15.5 (-22.6 to -8.3)
	p-value	<0.001		<0.001
	Cohen's *d*	0.55		0.50
Breathlessness[m]	Care as usual - mean	1.0		1.0
	Intervention - mean	0.7		0.6
	Difference (95 % CI)	-0.32 (-0.52 to -0.11)		-0.46 (-0.67 to -0.25)
	p-value	0.003		<0.001
	Cohen's *d*	0.36		0.56
Cognitive difficulties[n]	Care as usual - mean	3.2		3.2
	Intervention - mean	2.7		2.7

© 2024 Nerli TF et al. *JAMA Network Open.*

	Difference (95 % CI)	-0.50 (-0.73 to -0.27)		-0.42 (-0.65 to -0.18)
	p-value	<0.001		0.001
	Cohen's *d*	0.48		0.40
Sleep problems[o]	Care as usual - mean	38.5		36.9
	Intervention - mean	41.8		43.1
	Difference (95 % CI)	3.3 (1.3 to 5.3)		6.2 (4.0 to 8.4)
	p-value	0.001		<0.001
	Cohen's *d*	0.33		0.56
Anxiety symptoms[p]	Care as usual - mean	6.9		7.1
	Intervention - mean	5.6		5.8
	Difference (95 % CI)	-1.3 (-2.2 to -0.51)		-1.3 (-2.2 to -0.48)
	p-value	0.002		0.002
	Cohen's *d*	0.33		0.33
Depressive symptoms[q]	Care as usual - mean	6.3		6.2
	Intervention - mean	4.7		4.5
	Difference (95 % CI)	-1.7 (-2.5 to -0.82)		-1.6 (-2.5 to -0.75)
	p-value	<0.001		<0.001
	Cohen's *d*	0.42		0.42
Smell and/or taste abnormalities[r]	Care as usual - mean	1.9		1.9
	Intervention - mean	1.7		1.7
	Difference (95 % CI)	-0.16 (-0.40 to 0.09)		-0.20 (-0.46 to 0.06)
	p-value	0.21		0.13
	Cohen's *d*	0.12		0.15

© 2024 Nerli TF et al. *JAMA Network Open.*

Abbreviations: SF-36, Short Form Health Survey; CI, confidence interval; T1, first follow-up, after completion of the intervention (approximately 6 months after inclusion); T2, second follow-up, approximately 6 months after completion of the intervention (approximately 12 months after inclusion).

[a]Per protocol analyses were conducted as sensitivity analyses. For each statistical analysis, the net intervention effect (the mean change in the intervention group minus the mean change in the usual care group) was calculated from the parameters of the fitted ANCOVA model and reported with 95 % confidence interval. As sex had a somewhat skewed distribution across the two treatment allocation groups, this variable was included as a covariate in the ANCOVA modeling.

[b]Based upon 10 single items from the Short Form Health Survey[7]; total range 0-100 where higher scores imply better physical function.

[c]Based upon 4 single items from the Short Form Health Survey[7]; total range 0-100 where higher scores imply fewer limitations due to physical problems.

[d]Based upon 2 single items from the Short Form Health Survey[7]; total range 0-100 where higher scores imply less pain.

[e]Based upon 5 single items from the Short Form Health Survey[7]; total range 0-100 where higher scores imply better general health.

[f]Based upon 4 single items from the Short Form Health Survey[7]; total range 0-100 where higher scores imply better vitality.

[g]Based upon 2 single items from the Short Form Health Survey[7]; total range 0-100 where higher scores imply better social functioning.

[h]Based upon 3 single items from the Short Form Health Survey[7]; total range 0-100 where higher scores imply fewer limitations due to emotional problems.

[i]Based upon 5 single items from the Short Form Health Survey[7]; total range 0-100 where higher scores imply better mental health.

[j]Based upon 11 single items from the Return-to-work self-efficacy scale[13]; total range 1-6 where higher scores imply high work-related self-efficacy.

[k]Based upon 11 single items from the Chalder Fatigue Scale[6]; total range 0-33 where higher scores imply more fatigue.

[l]Based upon 5 single items from the DePaul Symptom Questionnaire[8] addressing frequency of symptoms; total range 0-100 where higher scores imply more post-exertional malaise.

[m]Based upon 1 single item from the Medical Research Council Dyspnoea Scale[9]; total range 0-4 where higher scores imply more dyspnea.

[n]Based upon the average of 4 single items addressing memory, concentration, confusion and ability to take decisions used in a previous Covid-19 cohort study[10]; total range 1-5 where higher scores imply more cognitive difficulties.

[o]Based upon 12 single items from the Karolinska Sleep Questionnaire[11] addressing frequency of sleep related problems; total range 12-72 where higher scores imply better sleep.

[p]Based upon 7 single items from the Hospital Anxiety and Depression Scale anxiety subscale[12]; total range 0-21 where higher scores imply more symptoms related to anxiety.

[q]Based upon 7 single items from the Hospital Anxiety and Depression Scale depression subscale[12]; total range 0-21 where higher scores imply more symptoms related to depression.

[r]Based upon the average of 2 single items addressing smell and taste abnormalities, respectively, used in a previous Covid-19 cohort study[10]; total range 1-5 where higher scores imply more smell/taste abnormalities.

© 2024 Nerli TF et al. *JAMA Network Open.*

eTable 7. Sensitivity analysis 2.

**Outcome of the intervention. Ordinal logistic regression[a] of selected variables, per protocol-analyses**			
		**T1 (n=224)**	**T2 (n=224)**
Secondary endpoints, SF-36 subscores			
Role limitations due to physical problems[b]	OR (95 % CI)	2.0 (1.1 to 3.6)	2.7 (1.5 to 4.8)
	p-value	0.02	0.001
Role limitations due to emotional problems[c]	OR (95 % CI)	1.4 (0.8 to 2.3)	2.5 (1.4 to 4.2)
	p-value	0.20	0.001
Secondary endpoints, symptoms			
Breathlessness[d]	OR (95 % CI)	0.55 (0.33 to 0.89)	0.34 (0.20 to 0.58)
	p-value	0.02	<0.001
Smell and/or taste abnormalities[e]	OR (95 % CI)	0.67 (0.40 to 1.12)	0.67 (0.38 to 1.17)
	p-value	0.13	0.16

Abbrevations: SF-36, Short Form Health Survey; T1, first follow-up, after completion of the intervention (approximately 6 months after inclusion); T2, second follow-up, approximately 6 months after completion of the intervention (approximately 12 months after inclusion); OR, Odds ratio; CI, confidence interval.
[a]The care as usual-group is the reference category.
[b]Based upon 4 single items from the Short Form Health Survey[7]; total range 0-100 where higher scores imply fewer limitations due to physical problems. The results were distributed non-normally across five categories, and the variable was therefore selected for sensitivity analyses featuring ordinal logistic regression.
[c]Based upon 3 single items from the Short Form Health Survey[7]; total range 0-100 where higher scores imply fewer limitations due to emotional problems. The results were distributed non-normally across four categories, and the variable was therefore selected for sensitivity analyses featuring ordinal logistic regression.
[d]Based upon 1 single item from the Medical Research Council Dyspnoea Scale[9]; total range 0-4 where higher scores imply more dyspnea. The results were distributed non-normally, and the variable was therefore selected for sensitivity analyses featuring ordinal logistic regression.
[e]Based upon the average of 2 single items addressing smell and taste abnormalities, respectively, used in a previous Covid-19 cohort study[10]; total range 1-5 where higher scores imply more smell/taste abnormalities. The results were distributed non-normally across 9 categories, and the variable was therefore selected for sensitivity analyses featuring ordinal logistic regression.

© 2024 Nerli TF et al. *JAMA Network Open.*

eTable 8. Participants meeting recovery threshold[a] of self-reported physical function, per-protocol data.

	T1				T2		
	CAU	Intervention	p-value[b]		CAU	Intervention	p-value[b]
**Non-recovery**	72 (68)	63 (50)	0.007		71 (70)	55 (44)	<0.001
**Recovery**	43 (32)	62 (50)			31 (30)	70 (56)	

Abbreviations: CAU, care as usual; SF-36, Short Form Health Survey; T1, first follow-up, after completion of the intervention (approximately 6 months after inclusion); T2, second follow-up, approximately 6 months after completion of the intervention (approximately 12 months after inclusion)
[a]Recovery threshold was defined as a SF-36 Physical Functioning Subscale score at the population norm[14] (~85) or higher.
[b]Chi-square test, 2-sided.

© 2024 Nerli TF et al. *JAMA Network Open.*

eTable 9. Subgroup analyses according to PIFS caseness[10,15] at inclusion. Intention-to-treat analysis[a].

		T1 (n=314)			T2 (n=314)	
		PIFS cases (n=108)	Non-PIFS cases (n=206)		PIFS cases (n=108)	Non-PIFS cases (n=206)
Primary endpoint, SF-36 subscore						
Physical function[b]	Care as usual - mean	60.2	76.3		60.8	76.6
	Intervention - mean	71.0	84.6		72.5	84.2
	Difference (95 % CI)	10.8 (3.4 to 18.2)	8.3 (2.4 to 14.3)		11.7 (3.7 t0 19.6)	7.6 (1.7 to 13.4)
	p-value	0.004	0.007		0.004	0.012
	Cohen's *d*	0.57	0.40		0.56	0.37
	p-value for interaction PIFS caseness*treatment allocation	0.80			0.94	
Secondary endpoints, SF-36 subscores						
Role limitations due to physical problems[c]	Care as usual - mean	11.2	27.6		16.6	28.8
	Intervention - mean	20.9	37.3		33.8	42.3
	Difference (95 % CI)	9.7 (-4.8 to 24.2)	9.7 (-3.0 to 22.5)		17.2 (1.0 to 33.4)	13.4 (-1.2 to 28.1)
	p-value	0.19	0.14		0.04	0.07
	Cohen's *d*	0.31	0.23		0.46	0.31
	p-value for interaction PIFS caseness*treatment allocation	0.10			0.44	
Bodily pain[d]	Care as usual - mean	51.2	51.6		49.6	52.6
	Intervention - mean	51.5	51.5		53.2	54.3
	Difference (95 % CI)	0.3 (-4.4 to 3.0)	-0.1 (-3.8 to 3.7)		3.6 (-1.6 to 8.7)	1.8 (-2.2 to 5.7)

© 2024 Nerli TF et al. *JAMA Network Open.*

	p-value	0.91	0.98	0.17	0.37
	Cohen's *d*	0.03	0.00	0.32	0.17
	p-value for interaction PIFS caseness*treatment allocation	0.98		0.21	
General health[e]	Care as usual - mean	42.6	46.2	39.6	47.1
	Intervention - mean	49.8	56.8	48.3	54.3
	Difference (95 % CI)	7.2 (-1.6 to 16.0)	10.6 (3.5 to 17.7)	8.7 (-0.01 to 17.4)	7.2 (-0.7 to 15.1)
	p-value	0.11	0.004	0.05	0.07
	Cohen's *d*	0.33	0.44	0.38	0.28
	p-value for interaction PIFS caseness*treatment allocation	0.42		0.10	
Vitality[f]	Care as usual - mean	30.6	35.4	24.9	32.6
	Intervention - mean	36.6	42.5	34.3	39.3
	Difference (95 % CI)	6.1 (-2.1 to 14.3)	7.0 (0.2 to 13.9)	9.4 (1.0 to 17.8)	6.8 (0.2 to 13.3)
	p-value	0.15	0.04	0.03	0.04
	Cohen's *d*	0.31	0.32	0.48	0.32
	p-value for interaction PIFS caseness*treatment allocation	0.24		0.42	
Social functioning[g]	Care as usual - mean	44.1	47.0	43.4	57.7
	Intervention - mean	59.0	61.2	63.9	68.4
	Difference (95 % CI)	14.9 (3.1 to 26.7)	14.2 (5.7 to 22.7)	20.5 (10.0 to 31.1)	10.7 (2.4 to 19.0)
	p-value	0.01	0.001	<0.001	0.01
	Cohen's *d*	0.56	0.50	0.78	0.39

© 2024 Nerli TF et al. *JAMA Network Open.*

	p-value for interaction PIFS caseness*treatment allocation	0.89			0.02	
Role limitations due to emotional problems[h]	Care as usual - mean	61.5	62.0		47.3	56.0
	Intervention - mean	61.2	63.7		70.4	70.3
	Difference (95 % CI)	-0.3 (-21.1 to 20.5)	1.7 (-14.7 to 18.1)		23.0 (3.5 to 32.6)	14.3 (-1.6 to 30.2)
	p-value	0.98	0.84		0.02	0.08
	Cohen's *d*	0.01	0.04		0.49	0.30
	p-value for interaction PIFS caseness*treatment allocation	0.87			0.49	
Mental health[i]	Care as usual - mean	66.3	66.9		64.3	67.3
	Intervention - mean	72.8	71.0		72.6	73.0
	Difference (95 % CI)	6.5 (0.8 to 12.3)	4.1 (-0.7 to 8.8)		8.2 (3.2 to 13.3)	5.7 (1.6 to 9.9)
	p-value	0.03	0.09		0.001	0.007
	Cohen's *d*	0.45	0.25		0.53	0.38
	p-value for interaction PIFS caseness*treatment allocation	0.66			0.55	
Secondary endpoint, working abilities						
Return to work self-efficacy[j]	Care as usual - mean	3.0	3.4		3.1	3.7
	Intervention - mean	3.5	3.8		3.7	4.0
	Difference (95 % CI)	0.5 (0.1 to 1.0)	0.4 (0.1 to 0.8)		0.7 (0.2 to 1.2)	0.3 (-0.04 to 0.7)
	p-value	0.02	0.02		0.01	0.09
	Cohen's *d*	0.44	0.33		0.52	0.24
	p-value for interaction PIFS caseness*treatment allocation	0.97			0.25	

© 2024 Nerli TF et al. *JAMA Network Open.*

Secondary endpoints, symptoms						
Fatigue[k]	Care as usual - mean	22.6	20.5		22.4	18.7
	Intervention - mean	20.1	18.2		18.9	16.9
	Difference (95 % CI)	-2.5 (-5.3 to 0.3)	-2.3 (-4.6 to 0.04)		-3.5 (-6.3 to -0.7)	-1.8 (-3.9 to 0.3)
	p-value	0.08	0.05		0.01	0.09
	Cohen's *d*	0.35	0.32		0.50	0.27
	p-value for interaction PIFS caseness*treatment allocation	0.63			0.08	
Post-exertional malaise[l]	Care as usual - mean	62.8	44.0		64.7	44.8
	Intervention - mean	46.7	33.3		48.2	34.5
	Difference (95 % CI)	-16.0 (-27.1 to -4.9)	-10.7 (-19.0 to -2.4)		-16.5 (-28.1 to -4.9)	-10.4 (-19.1 to -1.6)
	p-value	0.005	0.01		0.005	0.02
	Cohen's *d*	0.59	0.36		0.57	0.35
	p-value for interaction PIFS caseness*treatment allocation	0.21			0.13	
Breathlessness[m]	Care as usual - mean	1.3	0.8		1.4	0.8
	Intervention - mean	1.1	0.6		1.0	0.5
	Difference (95 % CI)	-0.2 (-0.6 to 0.1)	-0.2 (-0.5 to 0.1)		-0.4 (-0.8 to -0.1)	-0.4 (-0.7 to -0.1)
	p-value	0.21	0.20		0.03	0.009
	Cohen's *d*	0.27	0.21		0.46	0.43
	p-value for interaction PIFS caseness*treatment allocation	0.13			0.07	
Cognitive difficulties[n]	Care as usual - mean	3.5	3.2		3.3	2.9
	Intervention - mean	3.0	2.8		2.9	2.7

© 2024 Nerli TF et al. *JAMA Network Open.*

	Difference (95 % CI)	-0.5 (-0.9 to -0.2)	-0.4 (-0.7 to -0.1)		-0.4 (-0.8 to -0.006)	-0.2 (-0.5 to 0.05)
	p-value	0.006	0.02		0.05	0.10
	Cohen's *d*	0.54	0.33		0.37	0.23
	p-value for interaction PIFS caseness*treatment allocation	0.28			0.24	
Sleep problems[o]	Care as usual - mean	36.7	41.2		34.8	39.7
	Intervention - mean	39.1	42.9		40.9	43.9
	Difference (95 % CI)	2.4 (-1.1 to 5.9)	1.7 (-1.3 to 4.7)		6.1 (2.4 to 9.9)	4.2 (1.1 to 7.4)
	p-value	0.17	0.26		0.001	0.009
	Cohen's *d*	0.26	0.16		0.58	0.39
	p-value for interaction PIFS caseness*treatment allocation	0.32			0.27	
Anxiety symptoms[p]	Care as usual - mean	6.9	6.6		7.6	7.0
	Intervention - mean	5.6	6.1		6.1	6.3
	Difference (95 % CI)	-1.3 (-2.6 to 0.01)	-0.5 (-1.4 to 0.4)		-1.4 (-2.6 to -0.2)	-0.7 (-1.5 to 0.2)
	p-value	0.05	0.26		0.02	0.14
	Cohen's *d*	0.36	0.14		0.38	0.19
	p-value for interaction PIFS caseness*treatment allocation	0.85			0.41	
Depressive symptoms[q]	Care as usual - mean	6.4	5.9		7.1	5.6
	Intervention - mean	5.0	4.9		5.2	4.7
	Difference (95 % CI)	-1.5 (-2.7 to -0.2)	-1.0 (-1.8 to -0.1)		-1.9 (-3.3 to -0.6)	-0.8 (-1.7 to 0.04)
	p-value	0.03	0.02		0.005	0.06
	Cohen's *d*	0.39	0.29		0.49	0.25

© 2024 Nerli TF et al. *JAMA Network Open.*

	p-value for interaction PIFS caseness*treatment allocation	0.44			0.17	
Smell and/or taste abnormalities[r]	Care as usual - mean	2.3	1.7		2.2	1.9
	Intervention - mean	1.8	1.8		1.8	1.9
	Difference (95 % CI)	-0.5 (-1.0 to 0.03)	0.1 (-0.3 to 0.5)		-0.4 (-0.9 to 0.1)	-0.02 (-0.4 to 0.4)
	p-value	0.07	0.74		0.16	0.91
	Cohen's *d*	0.35	0.05		0.28	0.02
	p-value for interaction PIFS caseness*treatment allocation	0.16			0.85	

Abbreviations: PIFS, post-infective fatigue syndrome; SF-36, Short Form Health Survey; CI, confidence interval; T1, first follow-up, after completion of the intervention (approximately 6 months after inclusion); T2, second follow-up, approximately 6 months after completion of the intervention (approximately 12 months after inclusion).

[a]The analysis was an intention to treat analysis of all randomized participants featuring multiple imputation of missing values. For each statistical analysis, the net intervention effect (the mean change in the intervention group minus the mean change in the usual care group) was calculated from the parameters of the fitted ANCOVA model and reported with 95 % confidence interval.

[b]Based upon 10 single items from the Short Form Health Survey[7]; total range 0-100 where higher scores imply better physical function.

[c]Based upon 4 single items from the Short Form Health Survey[7]; total range 0-100 where higher scores imply fewer limitations due to physical problems.

[d]Based upon 2 single items from the Short Form Health Survey[7]; total range 0-100 where higher scores imply less pain.

[e]Based upon 5 single items from the Short Form Health Survey[7]; total range 0-100 where higher scores imply better general health.

[f]Based upon 4 single items from the Short Form Health Survey[7]; total range 0-100 where higher scores imply better vitality.

[g]Based upon 2 single items from the Short Form Health Survey[7]; total range 0-100 where higher scores imply better social functioning.

[h]Based upon 3 single items from the Short Form Health Survey[7]; total range 0-100 where higher scores imply fewer limitations due to emotional problems.

[i]Based upon 5 single items from the Short Form Health Survey[7]; total range 0-100 where higher scores imply better mental health.

[j]Based upon 11 single items from the Return-to-work self-efficacy scale[13]; total range 1-6 where higher scores imply high work-related self-efficacy.

[k]Based upon 11 single items from the Chalder Fatigue Scale[6]; total range 0-33 where higher scores imply more fatigue.

[l]Based upon 5 single items from the DePaul Symptom Questionnaire[8] addressing frequency of symptoms; total range 0-100 where higher scores imply more post-exertional malaise.

[m]Based upon 1 single item from the Medical Research Council Dyspnoea Scale[9]; total range 0-4 where higher scores imply more dyspnea.

[n]Based upon the average of 4 single items addressing memory, concentration, confusion and ability to take decisions used in a previous Covid-19 cohort study[10]; total range 1-5 where higher scores imply more cognitive difficulties.

[o]Based upon 12 single items from the Karolinska Sleep Questionnaire[11] addressing frequency of sleep related problems; total range 12-72 where higher scores imply better sleep.

[p]Based upon 7 single items from the Hospital Anxiety and Depression Scale anxiety subscale[12]; total range 0-21 where higher scores imply more symptoms related to anxiety.

[q]Based upon 7 single items from the Hospital Anxiety and Depression Scale depression subscale[12]; total range 0-21 where higher scores imply more symptoms related to depression.

[r]Based upon the average of 2 single items addressing smell and taste abnormalities, respectively, used in a previous Covid-19 cohort study[10]; total range 1-5 where higher scores imply more smell/taste abnormalities.

© 2024 Nerli TF et al. *JAMA Network Open.*

eTable 10. Subgroup analyses according to PEM score* at inclusion. Intention-to-treat analysis[a].

		T1 (n=314)			T2 (n=314)	
		High PEM (n=92)	Low PEM (n=222)		High PEM (n=92)	Low PEM (n=222)
Primary endpoint, SF-36 subscore						
Physical function[b]	Care as usual - mean	59.7	75.4		61.6	75.2
	Intervention - mean	73.3	82.7		71.1	83.8
	Difference (95 % CI)	13.7 (5.8 to 21.5)	7.3 (1.6 to 13.0)		9.5 (0.5 to 18.6)	8.6 (2.8 to 14.5)
	p-value	0.001	0.01		0.04	0.004
	Cohen's *d*	0.72	0.34		0.44	0.42
	p-value for interaction PEM score*treatment allocation	0.20			0.40	
Secondary endpoints, SF-36 subscores						
Role limitations due to physical problems[c]	Care as usual - mean	11.7	26.4		13.0	29.6
	Intervention - mean	15.9	37.8		23.4	45.7
	Difference (95 % CI)	4.1 (-12.0 to 20.3)	11.3 (-0.9 to 23.5)		10.4 (-4.7 to 25.6)	16.1 (1.9 to 30.3)
	p-value	0.62	0.07		0.18	0.03
	Cohen's *d*	0.13	0.28		0.31	0.37
	p-value for interaction PEM score*treatment allocation	0.14			0.11	
Bodily pain[d]	Care as usual - mean	48.7	52.6		48.0	53.1
	Intervention - mean	50.5	51.9		52.6	54.5
	Difference (95 % CI)	1.8 (-3.5 to 7.1)	-0.6 (-4.1 to 2.8)		4.6 (-0.4 to 9.7)	1.4 (-2.5 to 5.3)
	p-value	0.51	0.72		0.07	0.48
	Cohen's *d*	0.16	0.06		0.43	0.13

© 2024 Nerli TF et al. *JAMA Network Open.*

	p-value for interaction PEM score*treatment allocation	0.16			0.03	
General health[e]	Care as usual - mean	38.5	47.8		36.1	48.3
	Intervention - mean	50.0	56.0		44.4	55.2
	Difference (95 % CI)	11.5 (2.4 to 20.6)	8.2 (1.1 to 15.3)		8.3 (-1.2 to 17.8)	6.9 (-0.7 to 14.5)
	p-value	0.01	0.02		0.09	0.08
	Cohen's *d*	0.49	0.36		0.33	0.28
	p-value for interaction PEM score*treatment allocation	0.18			0.06	
Vitality[f]	Care as usual - mean	24.6	37.7		24.5	32.2
	Intervention - mean	34.0	43.1		29.7	40.7
	Difference (95 % CI)	9.3 (-0.2 to 18.8)	5.4 (-0.7 to 11.6)		5.2 (-3.4 to 13.9)	8.5 (2.2 to 14.7)
	p-value	0.05	0.09		0.24	0.009
	Cohen's *d*	0.45	0.26		0.26	0.42
	p-value for interaction PEM score*treatment allocation	0.06			0.40	
Social functioning[g]	Care as usual - mean	38.4	49.2		42.7	57.1
	Intervention - mean	53.8	63.1		55.6	71.3
	Difference (95 % CI)	15.3 (3.7 to 27.0)	13.9 (5.7 to 22.2)		12.9 (1.6 to 24.3)	14.2 (6.3 to 22.0)
	p-value	0.01	0.001		0.03	<0.001
	Cohen's *d*	0.59	0.50		0.49	0.54
	p-value for interaction PEM score*treatment allocation	0.41			0.07	
Role limitations due to emotional problems[h]	Care as usual - mean	47.7	68.0		49.4	54.5
	Intervention - mean	44.9	69.8		63.2	73.2
	Difference (95 % CI)	-2.8 (-25.6 to 19.9)	1.8 (-14.1 to 17.8)		13.8 (-6.5 to 34.0)	18.7 (3.0 to 34.3)

© 2024 Nerli TF et al. *JAMA Network Open.*

	p-value	0.81	0.82		0.18	0.02
	Cohen's *d*	0.06	0.04		0.29	0.39
	p-value for interaction PEM score*treatment allocation	0.08			0.92	
Mental health[i]	Care as usual - mean	61.3	68.9		59.9	69.0
	Intervention - mean	67.1	73.5		67.6	75.0
	Difference (95 % CI)	5.7 (-1.2 to 12.6)	4.6 (0.3 to 8.8)		7.7 (1.9 to 13.5)	6.0 (2.0 to 10.0)
	p-value	0.10	0.04		0.01	0.003
	Cohen's *d*	0.36	0.29		0.49	0.42
	p-value for interaction PEM score*treatment allocation	0.38			0.09	
Secondary endpoint, working abilities						
Return to work self-efficacy[j]	Care as usual - mean	2.7	3.5		3.0	3.6
	Intervention - mean	3.1	3.9		3.4	4.1
	Difference (95 % CI)	0.4 (-0.1 to 0.9)	0.5 (0.1 to 0.8)		0.4 (-0.1 to 0.9)	0.4 (0.1 to 0.8)
	p-value	0.09	0.005		0.16	0.02
	Cohen's *d*	0.33	0.38		0.31	0.34
	p-value for interaction PEM score*treatment allocation	0.12			0.25	
Secondary endpoints, symptoms						
Fatigue[k]	Care as usual - mean	23.8	20.2		23.5	18.5
	Intervention - mean	20.5	18.1		20.1	16.6
	Difference (95 % CI)	-3.3 (-6.2 to -0.3)	-2.1 (-4.4 to 0.2)		-3.4 (-6.4 to -0.5)	-2.0 (-4.1 to 0.2)
	p-value	0.03	0.07		0.03	0.07
	Cohen's *d*	0.50	0.29		0.49	0.30
	p-value for interaction PEM score*treatment allocation	0.22			0.01	

© 2024 Nerli TF et al. *JAMA Network Open.*

Post-exertional malaise[l]	Care as usual - mean	70.4	42.0		70.2	43.9
	Intervention - mean	54.5	31.1		58.8	31.3
	Difference (95 % CI)	-15.9 (-28.1 to -3.8)	-10.9 (-19.0 to -2.8)		-11.5 (-23.0 to 0.8)	-12.6 (-21.2 to -4.0)
	p-value	0.01	0.008		0.07	0.004
	Cohen's *d*	0.61	0.39		0.41	0.45
	p-value for interaction PEM score*treatment allocation	0.18			0.26	
Breathlessness[m]	Care as usual - mean	1.3	0.8		1.4	0.9
	Intervention - mean	1.0	0.6		1.0	0.5
	Difference (95 % CI)	-0.2 (-0.7 to 0.2)	-0.2 (-0.5 to 0.1)		-0.4 (-0.8 to 0.002)	-0.4 (-0.6 to -0.1)
	p-value	0.29	0.21		0.05	0.01
	Cohen's *d*	0.26	0.21		0.42	0.42
	p-value for interaction PEM score*treatment allocation	0.19			0.09	
Cognitive difficulties[n]	Care as usual - mean	3.8	3.1		3.6	2.8
	Intervention - mean	3.1	2.8		3.0	2.7
	Difference (95 % CI)	-0.7 (-1.1 to -0.3)	-0.3 (-0.6 to -0.01)		-0.6 (-1.0 to -0.2)	-0.2 (-0.5 to 0.1)
	p-value	<0.0001	0.04		0.005	0.26
	Cohen's *d*	0.76	0.28		0.56	0.17
	p-value for interaction PEM score*treatment allocation	0.04			0.03	
Sleep problems[o]	Care as usual - mean	34.7	41.7		32.6	40.3
	Intervention - mean	37.8	43.1		38.1	44.8
	Difference (95 % CI)	3.1 (-0.6 to 6.8)	1.4 (-1.5 to 4.2)		5.4 (1.6 to 9.2)	4.5 (1.3 to 7.6)
	p-value	0.10	0.35		0.005	0.006
	Cohen's *d*	0.32	0.14		0.53	0.44

© 2024 Nerli TF et al. *JAMA Network Open.*

	p-value for interaction PEM score*treatment allocation	0.06			0.05	
Anxiety symptoms[p]	Care as usual - mean	8.4	6.0		8.9	6.4
	Intervention - mean	6.9	5.5		7.8	5.6
	Difference (95 % CI)	-1.5 (-3.0 to -0.003)	-0.4 (-1.2 to 0.4)		-1.1 (-2.6 to 0.3)	-0.8 (-1.6 to -0.1)
	p-value	0.05	0.31		0.13	0.04
	Cohen's *d*	0.37	0.13		0.29	0.26
	p-value for interaction PEM score*treatment allocation	0.005			0.005	
Depressive symptoms[q]	Care as usual - mean	7.1	5.7		7.4	5.5
	Intervention - mean	5.9	4.5		6.3	4.3
	Difference (95 % CI)	-1.2 (-2.5 to 0.1)	-1.2 (-2.0 to -0.3)		-1.1 (-2.6 to 0.4)	-1.2 (-2.1 to -0.4)
	p-value	0.08	0.005		0.15	0.004
	Cohen's *d*	0.36	0.33		0.30	0.38
	p-value for interaction PEM score*treatment allocation	0.82			0.28	
Smell and/or taste abnormalities[r]	Care as usual - mean	2.1	1.8		2.2	1.9
	Intervention - mean	2.1	1.7		2.2	1.7
	Difference (95 % CI)	0.002 (-0.5 to 0.5)	-0.2 (-0.5 to 0.2)		-0.03 (-0.6 to 0.5)	-0.2 (-0.5 to 0.2)
	p-value	1.0	0.40		0.90	0.36
	Cohen's *d*	0.00	0.12		0.02	0.12
	p-value for interaction PEM score*treatment allocation	0.96			0.95	

© 2024 Nerli TF et al. *JAMA Network Open.*

Abbreviations: PEM, post-exertional malaise; SF-36, Short Form Health Survey; CI, confidence interval; T1, first follow-up, after completion of the intervention (approximately 6 months after inclusion); T2, second follow-up, approximately 6 months after completion of the intervention (approximately 12 months after inclusion).
*High-PEM is defines as the upper quartile and low-PEM is defined as the three lower quartiles.
[a]The analysis was an intention to treat analysis of all randomized participants featuring multiple imputation of missing values. For each statistical analysis, the net intervention effect (the mean change in the intervention group minus the mean change in the usual care group) was calculated from the parameters of the fitted ANCOVA model and reported with 95 % confidence interval.
[b]Based upon 10 single items from the Short Form Health Survey[7]; total range 0-100 where higher scores imply better physical function.
[c]Based upon 4 single items from the Short Form Health Survey[7]; total range 0-100 where higher scores imply fewer limitations due to physical problems.
[d]Based upon 2 single items from the Short Form Health Survey[7]; total range 0-100 where higher scores imply less pain.
[e]Based upon 5 single items from the Short Form Health Survey[7]; total range 0-100 where higher scores imply better general health.
[f]Based upon 4 single items from the Short Form Health Survey[7]; total range 0-100 where higher scores imply better vitality.
[g]Based upon 2 single items from the Short Form Health Survey[7]; total range 0-100 where higher scores imply better social functioning.
[h]Based upon 3 single items from the Short Form Health Survey[7]; total range 0-100 where higher scores imply fewer limitations due to emotional problems.
[i]Based upon 5 single items from the Short Form Health Survey[7]; total range 0-100 where higher scores imply better mental health.
[j]Based upon 11 single items from the Return-to-work self-efficacy scale[13]; total range 1-6 where higher scores imply high work-related self-efficacy.
[k]Based upon 11 single items from the Chalder Fatigue Scale[6]; total range 0-33 where higher scores imply more fatigue.
[l]Based upon 5 single items from the DePaul Symptom Questionnaire[8] addressing frequency of symptoms; total range 0-100 where higher scores imply more post-exertional malaise.
[m]Based upon 1 single item from the Medical Research Council Dyspnoea Scale[9]; total range 0-4 where higher scores imply more dyspnea.
[n]Based upon the average of 4 single items addressing memory, concentration, confusion and ability to take decisions used in a previous Covid-19 cohort study[10]; total range 1-5 where higher scores imply more cognitive difficulties.
[o]Based upon 12 single items from the Karolinska Sleep Questionnaire[11] addressing frequency of sleep related problems; total range 12-72 where higher scores imply better sleep.
[p]Based upon 7 single items from the Hospital Anxiety and Depression Scale anxiety subscale[12]; total range 0-21 where higher scores imply more symptoms related to anxiety.
[q]Based upon 7 single items from the Hospital Anxiety and Depression Scale depression subscale[12]; total range 0-21 where higher scores imply more symptoms related to depression.
[r]Based upon the average of 2 single items addressing smell and taste abnormalities, respectively, used in a previous Covid-19 cohort study[10]; total range 1-5 where higher scores imply more smell/taste abnormalities.

© 2024 Nerli TF et al. *JAMA Network Open.*

eTable 11. Adverse events[a]

Care as usual			Intervention	
AE	SAE[b]		AE	SAE
Diagnosed with postural orthostatic tachycardiac syndrome (POTS)	Hypertension and diabetes type 2, admitted to hospital		Diagnosed with AD/HD	Diabetes type 1 and hyperglycemia, admitted to hospital
Diagnosed with chronic obstructive pulmonary disease	Acute appendicitis, admitted to hospital		Spontaneous miscarriage	Acute appendicitis, admitted to hospital
Diagnosed with eye migraine	In need of detox stay, admitted to institution		One episode of urticaria	Basal cell carcinoma, removed surgically
Diagnosed with irritatable bowel syndrome	Acute pericarditis, admitted to hospital		One short episode of benign paroxysmal positional vertigo	Acute chest pain, admitted to hospital
Diagnosed with post commotio syndrome	Major depression, received electroconvulsive therapy		Antibiotics for erythema migrans	
Nose fracture	Acute ileus, admitted to hospital		Diagnosed with carpal tunnel syndrome	
Diagnosed with Diabetes type 2			One episode of pericoronitis	
Antibiotics for suspected Lyme disease				

Abbrevatioens: AE, adverse events; SAE, serious adverse events; AD/HD, attention deficit hyperactivity disorder.
[a]AE was defined as any medical occurrence in the follow-up period, whether or not attributed to or considered to be causally related to the intervention.
[b]SAE was defined as an AE meeting any of the following criteria: Deadly; life threating; required hospitalization; resulted in significant disability.

© 2024 Nerli TF et al. *JAMA Network Open.*

eTable 12. Decrease in SF-36 Physical Function Subscore from baseline to T1 in the intervention group, qualitative evaluation[a].

Patient	Decrease in subscore from T0-T1	Number of consultations	Qualitative description
1	-10	3	Video only. No common ground for combined effort, ended therapy early. Further decrease from T1-T2.
2	-10	8	Bad experience from before with “our” way of working, Symptom focused. Applied for permanent social benefits. Difficulties at work. Increase above baseline at T2.
3	-5	4	Described positive development, but challenges regarding family. Pregnant. Increase above baseline at T2.
4	-5	8	Multiple new infections before end of treatment. Experiencing work as a negative factor. Increase above baseline at T2.
5	-45	5	Increased symptoms after meeting with the medical doctor. Wanted more tests and examinations, and declined further treatment because of this. Same score T2.
6	-25	5	Long time since acute infection. Vaccine reaction. Applied for permanent social benefits and had ongoing insurance case with the government. Referred to Mental Health Care because of trauma at work during the pandemic. Further decrease from T1-T2.
7	-25	3	Disappointed with our treatment, ended follow-up in the second meeting with therapist. Further decrease from T1-T2.
8	-15	5	Effect of treatment and permanently back to work at encounter four. New infection and menopause before end of treatment. Increase again from T1-T2.
9	-10	4	Severely ill. Previous treatments without effect. Ended treatment at third encounter with therapist. Same score at T2.

Abbrevations: SF-36, Short Form Health Survey; T0, inclusion timepoint; T1, first follow-up, after completion of the intervention (approximately 6 months after inclusion); T2, second follow-up, approximately 6 months after completion of the intervention (approximately 12 months after inclusion).
[a]Qualitative evaluation by reading of journals from the Division of Physical Medicine and Rehabilitation, Vestfold Hospital Trust, written during the intervention period.

© 2024 Nerli TF et al. *JAMA Network Open.*

eTable 13. Increase in post exertional malaise with DePaul Symptom Questionnaire[8] addressing frequency of symptoms from baseline to T1 in the intervention group, qualitative evaluation[a]

Patient	Increase in subscore from T0-T1	Number of consultations	Qualitative description
1	5	5	Fibromyalgia. Applied for permanent social benefits. Child with congenital illness. Repeated infections towards the end of treatment. Decrease under baseline at T2.
2	50	5	Increased symptoms after meeting with the medical doctor. Wanted more tests and examinations, and declined further treatment because of this. Decrease again from T1-T2.
3	5	7	Severe symptoms, severe anxiety. Stomach pain and started new examinations because of this. Reinfected with covid-19 right before end of treatment. Decrease under baseline at T2.
4	15	3	In the last consultation the patient told us about a mental breakdown, which is only described as because of complex reasons. The patient did not meet for the next consultations. Decrease again from T1-T2.
5	15	7	On sick leave before start of treatment because of low back pain. Reinfection. Described as being ready for work, but after a long travel over continents with the family abroad it became worse of unknown reasons. Same score at T2.
6	10	8	Health anxiety, afraid of dementia. Neuropsychologist and MRI of the head with negative results. Revealed a traumatic childhood during the consultations, difficulties in the relationship with family members and negative working environment. Wanted to apply for permanent social benefits. No score at T2.
7	25	8	Bad experience from before with "our" way of working, Symptom focused. Applied for permanent social benefits. Difficulties at work. Same score at T2
8	10	3	Only smell and taste difficulties, affected quality of life for the patient. Practical advice for retraining smell and taste. Protocol deviation because the patient did not follow up appointments. Decrease again from T1-T2.
9	10	4	Only smell and taste difficulties. We never understood that there might have been other problems. We only gave practical advice regarding smell and taste. Further increase from T1-T2.
10	35	5	Effect of treatment and permanently back to work at encounter four. New infection and menopause before end of treatment. Decrease again from T1-T2.
11	30	3	Experienced effect of treatment, back to work 50%. From the journals, it is hard to understand why the patient scored worse on post-exertional malaise. Further increase from T1-T2.

© 2024 Nerli TF et al. *JAMA Network Open.*

12	15	6	Wanted specific advice on training, which we provided. Effect described in journals, but the patient was under examination for narrow coronary vessels towards the end of treatment. Decrease under baseline at T2.
13	10	3	Disappointed with our treatment, ended follow-up in the second meeting with therapist. Further increase from T1-T2.
14	20	8	Different expression of symptoms than everyone else, constant repeating pattern of symptoms without exceptions and no change in the condition during follow up. Did not want to accept our theoretical framework, and wanted to wait for a potential medical treatment further down the line. Still, both the patient ant therapist worked hard together, but no common ground was found even after 8 sessions. Same score at T2.

Abbrevations: T0, inclusion timepoint; T1, first follow-up, after completion of the intervention (approximately 6 months after inclusion); T2, second follow-up, approximately 6 months after completion of the intervention (approximately 12 months after inclusion).
[a]Qualitative evaluation by reading of journals from the Division of Physical Medicine and Rehabilitation, Vestfold Hospital Trust, written during the intervention period.

© 2024 Nerli TF et al. *JAMA Network Open.*

eFigure 1. Adherence to the modified Fukuda-definition of PIFS caseness.[10,15]

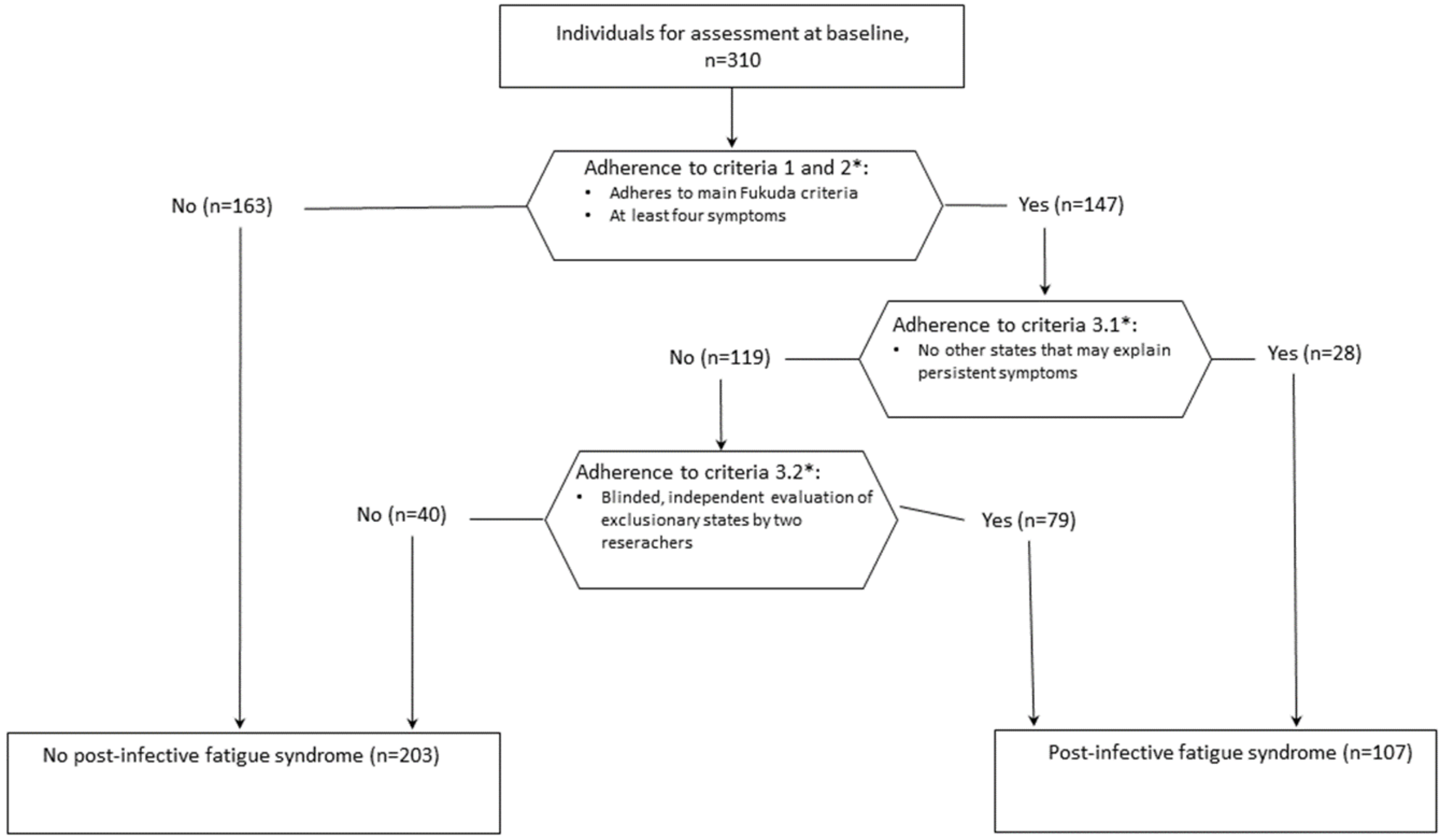

*Please see Statistical Analysis plan: Appendix, for table containing operationalization criteria.

© 2024 Nerli TF et al. *JAMA Network Open.*

eFigure 2. Correlation (Spearman's Rho) heatmap of symptom-endpoints and selected symptoms.

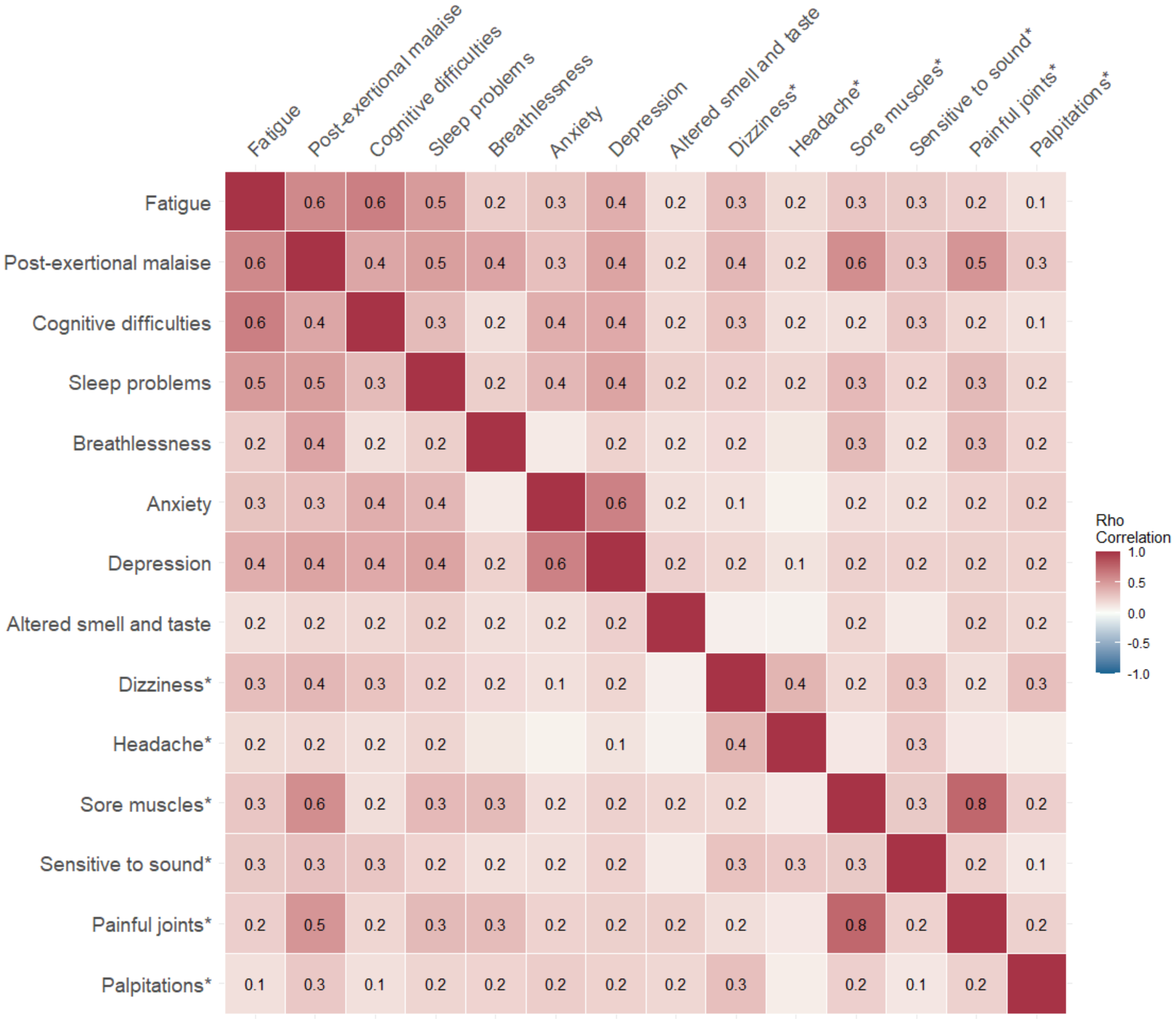

© 2024 Nerli TF et al. *JAMA Network Open.*

Non-significant (p<0.5) correlations have been left omitted from the figure for clarity.

*Individual symptoms with more than 50% prevalence (Table S2) and that were not encompassed by the symptom-endpoints, have been included in the figure

© 2024 Nerli TF et al. *JAMA Network Open.*

eFigure 3. Physical function score[a] (0-100) and trajectories[b] of participants who attended any follow-up timepoint[c].

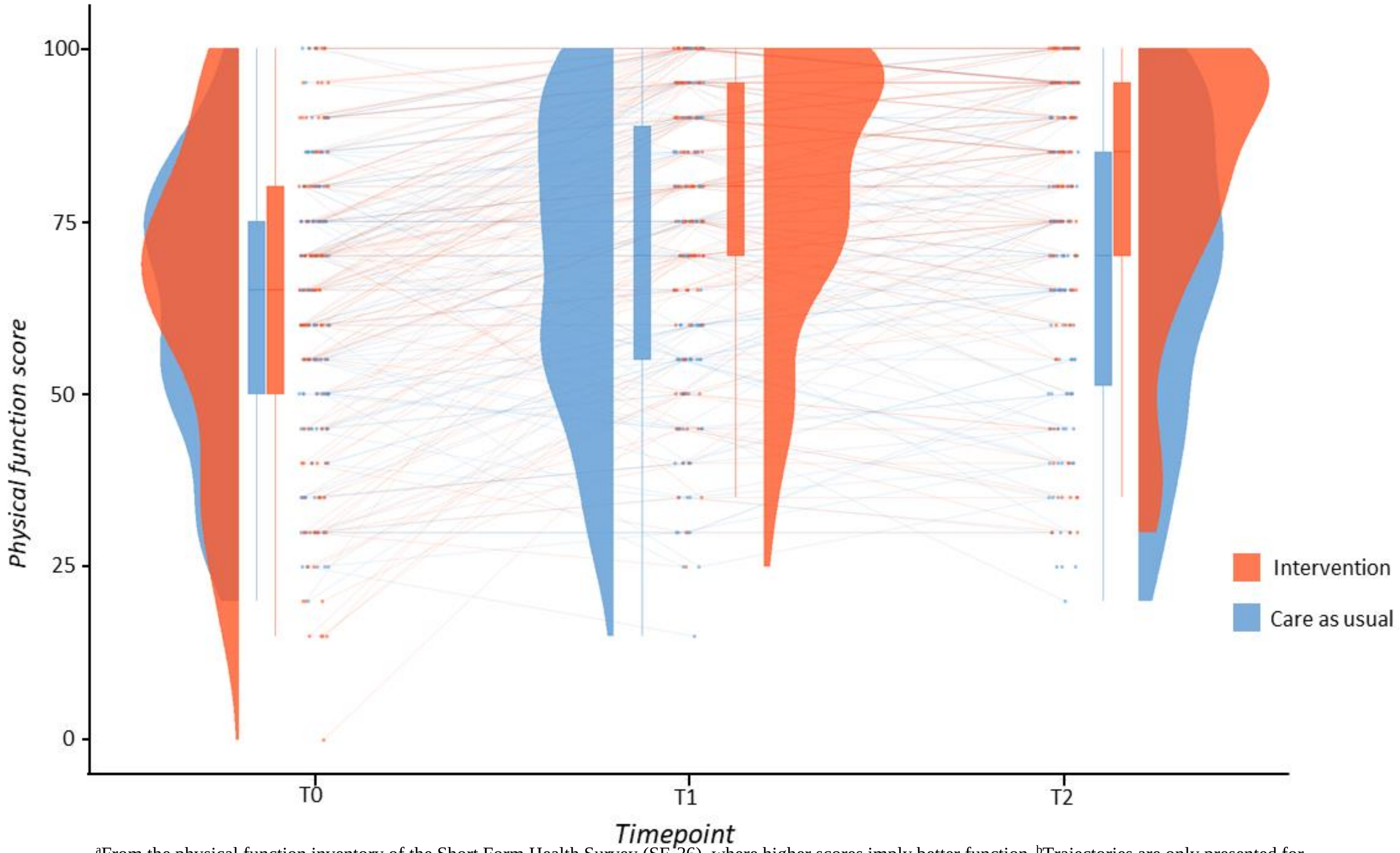

[a]From the physical function inventory of the Short Form Health Survey (SF-36), where higher scores imply better function. [b]Trajectories are only presented for individuals attending T1. [c]N = 134 in the intervention group and N = 119 in the care-as-usual group attended follow-up at T1 and/or T2 in the per protocol data set. *Figure created using the ggrain package for R.*[16]

© 2024 Nerli TF et al. *JAMA Network Open.*

eReferences

1. Ursin H, Eriksen HR. Cognitive activation theory of stress (CATS). Neurosci Biobehav Rev. May 2010;34(6):877-81. doi:10.1016/j.neubiorev.2009.03.001
2. 23. Pincus T, Holt N, Vogel S, et al. Cognitive and affective reassurance and patient outcomes in primary care: a systematic review. Pain. Nov 2013;154(11):2407-2416. doi:10.1016/j.pain.2013.07.019
3. Munk A, Reme SE, Jacobsen HB. What Does CATS Have to Do With Cancer? The Cognitive Activation Theory of Stress (CATS) Forms the SURGE Model of Chronic Post-surgical Pain in Women With Breast Cancer. Front Psychol. 2021;12:630422. doi:10.3389/fpsyg.2021.630422
4. Kazantzis N, Beck JS, Clark DA, et al. Socratic Dialogue and Guided Discovery in Cognitive Behavioral Therapy: A Modified Delphi Panel. Int J Cogn Ther. 2018/06/01 2018;11(2):140-157. doi:10.1007/s41811-018-0012-2
5. Kazantzis N, Miller AR. A Comprehensive Model of Homework in Cognitive Behavior Therapy. Cognit Ther Res. 2022/02/01 2022;46(1):247-257. doi:10.1007/s10608-021-10247-z
6. Chalder T, Berelowitz G, Pawlikowska T, et al. Development of a fatigue scale. J Psychosom Res. 1993;37(2):147-53. doi:10.1016/0022-3999(93)90081-p
7. Ware JE, Jr., Sherbourne CD. The MOS 36-item short-form health survey (SF-36). I. Conceptual framework and item selction. Med Care. Jun 1992;30(6):473-83.
8. 30. Bedree H, Sunnquist M, Jason LA. The DePaul Symptom Questionnaire-2: A Validation Study. Fatigue. 2019;7(3):166-179. doi:10.1080/21641846.2019.1653471
9. Bestall JC, Paul EA, Garrod R, Garnham R, Jones PW, Wedzicha JA. Usefulness of the Medical Research Council (MRC) dyspnoea scale as a measure of disability in patients with chronic obstructive pulmonary disease. Thorax. Jul 1999;54(7):581-6. doi:10.1136/thx.54.7.581
10. Selvakumar J, Havdal LB, Drevvatne M, et al. Prevalence and Characteristics Associated With Post–COVID-19 Condition Among Nonhospitalized Adolescents and Young Adults. *JAMA Network Open*. 2023;6(3):e235763-e235763. doi:10.1001/jamanetworkopen.2023.5763
11. Akerstedt T, Ingre M, Broman JE, Kecklund G. Disturbed sleep in shift workers, day workers, and insomniacs. Chronobiol Int. Apr 2008;25(2):333-48. doi:10.1080/07420520802113922
12. Zigmond AS, Snaith RP. The hospital anxiety and depression scale. Acta Psychiatr Scand. Jun 1983;67(6):361-70. doi:10.1111/j.1600-0447.1983.tb09716.x
13. Shaw WS, Reme SE, Linton SJ, Huang YH, Pransky G. 3rd place, PREMUS best paper competition: development of the return-to-work self-efficacy (RTWSE-19) questionnaire--psychometric properties and predictive validity. Scand J Work Environ Health. Mar 2011;37(2):109-19. doi:10.5271/sjweh.3139
14. Jacobsen EL, Bye A, Aass N, et al. Norwegian reference values for the Short-Form Health Survey 36: development over time. Qual Life Res. May 2018;27(5):1201-1212. doi:10.1007/s11136-017-1684-4
15. Hickie I, Davenport T, Wakefield D, et al. Post-infective and chronic fatigue syndromes precipitated by viral and non-viral pathogens: prospective cohort study. BMJ. Sep 16 2006;333(7568):575. doi:10.1136/bmj.38933.585764.AE
16. Allen M, Poggiali D, Whitaker K, Marshall TR, van Langen J, Kievit RA (2021). “Raincloud plots: a multi-platform tool for robust data visualization [version 2; peer review: 2 approved].” *Wellcome Open Research*, **4**(63). doi:10.12688/wellcomeopenres.15191.2

© 2024 Nerli TF et al. *JAMA Network Open.*
