# Supplementary material for: Brief Outpatient Rehabilitation Program for Post–COVID-19 Condition: A Randomized Clinical Trial
Source: JAMA Netw Open. 2024 Dec 19;7(12):e2450744. doi: 10.1001/jamanetworkopen.2024.50744 (PMC11659907; doi:10.1001/jamanetworkopen.2024.50744)
Supplement: Supplement 4. — Data Sharing Statement [file jamanetwopen-e2450744-s004.pdf]

Data Sharing Statement

Nerli. Brief Outpatient Rehabilitation Program for Post–COVID-19 Condition. *JAMA Netw Open*. Published December 18, 2024. doi:10.1001/jamanetworkopen.2024.50744

Data
Additional Information: Clinical Trials, NCT05196451, www.clinicaltrials.gov
Data available: Yes
Data types: Deidentified participant data
How to access data: v.b.b.wyller@medisin.uio.no
When available: With publication

Supporting Documents
Document types: None

Additional Information
Who can access the data: Researchers whose proposed use of the data has been approved.
Types of analyses: For scientific purpose
Mechanisms of data availability: After approval of a proposal
